# Wellington: a novel method for the accurate identification of digital genomic footprints from DNase-seq data

**DOI:** 10.1093/nar/gkt850

**Published:** 2013-09-25

**Authors:** Jason Piper, Markus C. Elze, Pierre Cauchy, Peter N. Cockerill, Constanze Bonifer, Sascha Ott

**Affiliations:** ^1^Warwick Systems Biology Centre, University of Warwick, Coventry, CV4 7AL, United Kingdom, ^2^School of Cancer Sciences, Institute of Biomedical Research, College of Medical and Dental Sciences, University of Birmingham, Birmingham, B15 2TT, United Kingdom, ^3^Department of Statistics, University of Warwick, Coventry, CV4 7AL, United Kingdom and ^4^School of Immunity and Infection, Institute of Biomedical Research, College of Medical and Dental Sciences, University of Birmingham, Birmingham, B15 2TT, United Kingdom

## Abstract

The expression of eukaryotic genes is regulated by *cis*-regulatory elements such as promoters and enhancers, which bind sequence-specific DNA-binding proteins. One of the great challenges in the gene regulation field is to characterise these elements. This involves the identification of transcription factor (TF) binding sites within regulatory elements that are occupied in a defined regulatory context. Digestion with DNase and the subsequent analysis of regions protected from cleavage (DNase footprinting) has for many years been used to identify specific binding sites occupied by TFs at individual *cis*-elements with high resolution. This methodology has recently been adapted for high-throughput sequencing (DNase-seq). In this study, we describe an imbalance in the DNA strand-specific alignment information of DNase-seq data surrounding protein–DNA interactions that allows accurate prediction of occupied TF binding sites. Our study introduces a novel algorithm, Wellington, which considers the imbalance in this strand-specific information to efficiently identify DNA footprints. This algorithm significantly enhances specificity by reducing the proportion of false positives and requires significantly fewer predictions than previously reported methods to recapitulate an equal amount of ChIP-seq data. We also provide an open-source software package, pyDNase, which implements the Wellington algorithm to interface with DNase-seq data and expedite analyses.

## INTRODUCTION

The correct tissue-specific and temporal function of the genome is tightly controlled by transcription factors (TFs) that recognise specific DNA sequences and regulate the expression of specific genes. However, they do not act as single molecules but interact with each other to form large multi-protein assemblies that act as platforms for the recruitment of members of the epigenetic regulatory machinery (1,2). One of the significant challenges facing gene regulation studies is the identification of sites where TFs are bound to specific genes in a specific regulatory context. Although previous studies have shown a direct link between the sequence as well as tissue specificity of a number of TFs and gene expression patterns (3,4), the mechanisms behind how defined DNA sequences and the assembly of TF complexes translate into global gene expression patterns remains to be fully understood.

Characterising TF binding sites (TFBSs) across the entire genome is a monumental task. It is estimated that the total number of TFs in the human genome number ∼1500, where several hundred of these may be active in a given cell type at any one time (5). Currently, the ‘gold standard’ for identifying occupied TFBSs in a given context uses chromatin immunoprecipitation paired with high-throughput sequencing (ChIP-seq) (6), which requires either a high-quality antibody or high cell numbers or alternatively epitope tagging. Although ChIP-seq has proven to be extremely powerful, it is not without limitations: It is only possible to characterise one TF per experiment, it cannot be used alone to differentiate between primary and secondary binding (7), and the protein binding regions of the genome identified by ChIP-seq are in the order of several hundred base pairs. Progress has been made in this respect with the advent of ChIP-exo (8), which increases resolution of ChIP-seq data to below 50 bp, but this method has yet to see widespread adoption.

Another widely used approach in gene regulation studies uses DNase I as a tool to identify DNase I Hypersensitive Sites (DHSs) within chromatin (1). DHSs represent open chromatin regions that are normally only accessible at sites of active regulatory elements such as transcriptional enhancers. The recent development of DNase-seq has allowed more comprehensive mapping of the active chromatin landscape than is possible with ChIP-seq (9). The specific patterns of DNase I cleavage within DHSs also provide additional information about regions of DNA that are bound by proteins and are thereby protected from DNase I digestion, a feature that has been exploited for many years to obtain information about DNA–protein interactions at specific genes (10,11). However, the genome-wide data gained from this method are not trivial to analyse. DHSs can occupy hundreds of base pairs, and the entire complement of such sites contains an intrinsically high number of different specific TFBSs (9).

Although analyses of DNase-seq data were originally confined to identifying DHSs by peak detection, there have recently been several advances in the analysis of the raw tag counts that correspond to DNase activity at base pair resolution. The first of these digital genomic footprinting (DGF) methods were developed in yeast, where tag counts were processed with a rank transformation and tested for depletions in reads corresponding to occupied TFBSs using a binomial test (12). Subsequently, the first DGF studies in mammalian cells used a machine-learning approach where the tag counts were truncated, smoothed and differentiated, followed by the supervised training of a Hidden Markov Model on the known TFBSs in the *FMR1* promoter. Viterbi decoding was then performed to provide binary classifications (bound or unbound) for every base in the genome (13). Although several sets of footprints for various cell types as well as the model parameters were published, a software implementation was not made available. Another machine-learning approach, CENTIPEDE, trains an unsupervised Bayesian mixture model on the raw tag counts surrounding all genomic occurrences of a specified motif of interest to predict the binding states of each motif occurrence (14); however, unlike the previous methods, it cannot make predictions at arbitrary genomic loci. A software implementation of the CENTIPEDE algorithm is available but requires data to be pre-processed by the user into non-standard formats. The ENCODE project (15) has produced the most comprehensive set of DGFs in human cells by performing high-sequencing depth DNase-seq experiments on a multitude of cell types, adapting their previous footprinting methodology (12) to human data through the use of a metric that calculates the ratio of DNase-seq tags within a binding site to those directly outside (the Footprint Occupancy Score) (7).

Using publically available DNase-seq data from the ENCODE project, we describe how the alignment direction of DNA fragments relative to the reference strand exhibits a characteristic strand imbalance in the patterns surrounding known protein–DNA binding sites. We introduce Wellington, a novel footprinting algorithm that uses this knowledge to identify protein–DNA interactions in DNase-seq data with increased performance over previous methods, by reducing the number of false positives in our predictions. Alongside this, we provide the pyDNase software package to interface with DNase-seq data to run the Wellington algorithm and accelerate development of further analysis methods for these data. pyDNase and Wellington form a complete tool chain that can be used to identify protein–DNA interactions in any DNase-seq experiment performed according to the ‘double-hit’ protocol (16). Finally, we compared the performance of the different footprinting methods on a single data set, which we hope will be useful to the community in their decision of how to approach DGF tasks.

## MATERIALS AND METHODS

### Data

Aligned double-hit DNase-seq data and genomic co-ordinates of DHSs (K562: wgEncodeUwDgfK562, HepG2: wgEncodeUwDgfHepg2, A549: wgEncodeUwDgfA549, SkMC: wgEncodeUwDgfSkmcAln) and PhyloP conservation (Vertebrate phyloP46way) scores were downloaded from the UCSC genome browser (17). K562 data corresponding to the original single-hit DNase-seq library preparation method (9) were downloaded from the Sequence Read Archive (accession SRS131306) and aligned to hg19 using bowtie 1.0.0 (18) with the command line parameters ‘-a -best -strata -v 2 -m 1’. ChIP-seq data were downloaded as peaks from the ENCODE project’s ChIP-seq studies (19); for track names, see Supplementary Table S1.

### The Wellington algorithm

To detect protein–DNA binding sites, we must characterise the activity of DNase I and define what we consider to be a footprint. It is known that the activity of DNase I is lower in regions of inaccessible chromatin owing to protection of cleavage by histones or protein–DNA interactions. DNase I activity is therefore higher in regions of open chromatin without a bound protein. Protein–DNA binding sites can be detected by searching for a characteristic depletion of DNase I cuts compared with a large number of cuts in the surrounding region of open chromatin that do not harbour bound proteins.

**Figure 1.**
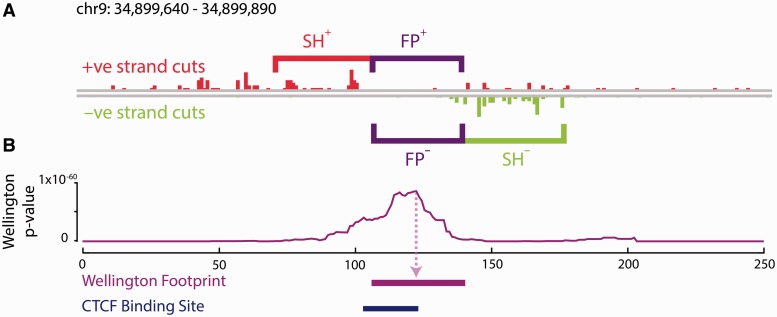
Wellington: a novel strand sensitive algorithm for the identification of protein–DNA binding sites from DNase-seq data. (**A**) The Wellington algorithm calculates *p*-values for every base pair in all DNase hypersensitive sites in a given DNase-seq data set, where the *s*-value is assigned to the base pair at the centre of the footprint. For each base pair, Wellington tests the hypothesis that there are significantly more reads aligning to the forward reference strand in the upstream shoulder region (SH^+^) with respect to the +ve strand footprint region (FP^+^) and significantly more reads aligning to the reverse reference strand in the downstream shoulder region (SH^–^) with respect to the −ve strand footprint region (FP^–^). (**B**) Example output of the Wellington algorithm. The corresponding footprint prediction recapitulates the ChIP-seq confirmed CTCF-binding site.

To formalise our hypothesis test, we use the notation introduced in Figure 1. We will call the region surrounding the possible footprint the shoulder region. Let l_FP_ be the length (in base pairs) of the possible footprint and l_SH_ be the length (in base pairs) of the shoulder on each side of the possible footprint. We consider counts of cuts in these regions where ‘cuts’ refers to 5′ ends of the aligned sequencing tags. We consider four cut counts: the total number of cuts on the forward reference strand inside the possible footprint (FP^+^), the cut count in the upstream shoulder region on the forward reference strand (SH^+^), the cut count on the backward reference strand inside the possible footprint (FP^−^) and the cut count in the downstream shoulder region on the backward reference strand (SH^−^).

We now test the null hypothesis that the number of reads is proportional to the region length by using a binomial test. As the number of reads can depend on the strand, e.g. because the protein structure might be such that it only inhibits DNase I activity on one strand, we test both strands separately. We consider these tests to be independent, as each ∼200 bp fragment will at most produce either one forward or one backward read close to the footprint site under investigation. With F(k, n, p) being the binomial cumulative distribution function, i.e. the probability of achieving at least k out of n successes with the probability of each success being p, we calculate a *p*-value using the formula *p*-value = {1-F[FP^+^, FP^++^ SH^+^, l_FP_/(l_FP_ + l_SH_)]} · {1-F[FP^−^, FP^−^ + SH^−^, l_FP_/(l_FP_ + l_SH_)]}. This *p*-value is for a given possible footprint of length l_FP_ with surrounding shoulder regions of length l_SH_.

We can calculate *p*-values for different possible footprint and shoulder lengths l_FP_ and l_SH_. We can then choose which regions we wish to consider footprints by selecting an appropriate threshold for the *p*-values and subsequently using a greedy selection strategy for footprint identification. The parameters l_FP_ and l_SH_ are individually determined for each footprint using maximum likelihood estimation. The default values for l_FP_ are bound between 11 and 26 base pairs, whereas l_SH_ is fixed at 35 base pairs. Both of these parameters can be user-settable at run time with either ranges or fixed values. Further details are provided in the supplementary material.

### Validation of predicted binding sites

We downloaded peaks determined by ENCODE’s peak calling algorithm (specifically, ENCODE’s ‘optimal’, high confidence set of peaks) for ChIP-seq experiments corresponding to a range of TFs. ChIP-seq confirmed binding sites were defined as motif instances falling within these peaks for each TF, and unbound motif locations were defined as motif instances falling outside ChIP-seq peaks.

To calculate ChIP-seq recapitulation, we used Wellington to calculate footprint *p*-values for each base pair in all DHSs and compared footprints with ChIP-seq positive motif instances. A ChIP-seq confirmed binding site is said to be successfully recapitulated by DNase-seq data if either at least 70% of the footprint is contained within the binding site or vice versa. This criterion is necessary as protection from DNase I is not always centred perfectly on a DNA motif. The same method was used when analysing Hesselberth *et al.* (12) footprints, Neph *et al.* (7) footprints and DHSs.

Average conservation scores were calculated using Vertebrate phyloP46way, and motif content was calculated using the genomic locations of 214 curated ChIP-seq verified position weight matrices published as part of the HOMER suite (20). For full details, see supplementary material.

## RESULTS

### Strand imbalance information increases the predictive power of footprinting algorithms

Strand-specific information in the context of DNase-seq data has been used primarily to describe TF-specific cleavage patterns in reference to the orientation of a known DNA motif (13,14). Previous efforts at predicting DGFs have been strand-agnostic, ignoring alignment strand information and considering DNase I cleavage activity as absolute, without regard to the orientation of the sequenced fragment relative to the cut site. However, if one considers that the DNA fragments generated by DNase cutting are likely to originate predominantly from within DHSs, with a high probability of spanning occupied binding sites, then the strand to which the sequence tags align is likely to be highly informative with regard to the relative position of TFBSs. This is because the upstream end of a DHS fragment will be aligned as a +ve strand sequence tag, whereas the downstream end will be aligned as a −ve strand sequence tag, as illustrated in Supplementary Figure S3. Hence, for DNA fragments that span DHSs, and encompass DNase I footprints, the DNase I cuts identified from +ve strand alignments will be concentrated to the left, and those from −ve strand alignments will be concentrated to the right. Chromatin structure influences the digestion pattern, as there is a lower probability of sequencing DNA fragments that extend away from the DHS. This is caused by the fact that these fragments will be of lower abundance due to the lower probability of generating a second DNase I cleavage within flanking regions occupied by nucleosomes. Such fragments will thus likely be discarded during the necessary process of size selection before or during library preparation.

**Figure 2.**
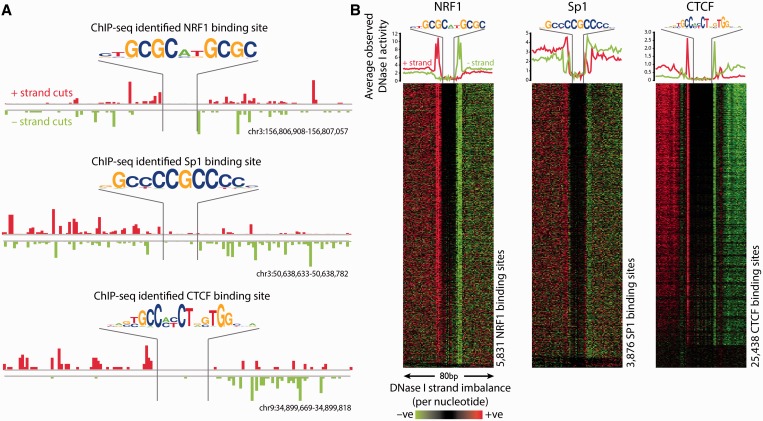
DNase I cleavage patterns surrounding known protein–DNA interactions as identified by ChIP-seq exhibit a strand imbalance, regardless of the strand where the binding motif is located. (**A**) Individual representative regions of DNase-seq data flanking NRF1, Sp1 and CTCF binding sites illustrate large numbers of sequencing fragments aligning to the positive reference strand upstream of the protein–DNA binding site and to the negative reference strand downstream of the protein–DNA binding site. These patterns exist independent of the direction in which the binding motif is located. (**B**) Heat maps show that the DNase-seq strand imbalance surrounding NRF1, Sp1 and CTCF binding sites identified by ChIP-seq exists on a genomic scale relative to the reference strand, irrespective of motif orientation (heat maps relative to motif orientation are shown in Supplementary Figure S4). Red indicates an excess of positive strand cuts over negative strand cuts per nucleotide position, and green indicates an excess of negative strand cuts. Binding sites are sorted from top to bottom in order of decreasing Footprint Occupancy Score (7).

We tested the aforementioned predictions by considering the alignment strand when visualising DNase I cleavage sites in the vicinity of known motifs using published DNase-seq data at ChIP-seq verified binding sites from K562 cells that are available from ENCODE (Figure 2A and B). Similar to the imbalance of sequencing reads observed in ChIP-seq and DHS mapping (21), we noted that DNase-seq data surrounding binding sites often exhibit an abundance of sequencing reads aligning to the +ve reference strand upstream of the binding site, and reads aligning to the −ve reference strand downstream of the binding site, consistent with these tags representing opposite ends of DNA fragments spanning protected regions. This was particularly evident when DNase I cuts at binding motifs for specific factors across the genome were collapsed into a heat map (Figure 2B). When investigating a diverse set of TFs, we noticed that the imbalance varies in strength, with some binding sites having diminished strand imbalance, and others showing almost none. However, we never observe a ‘reverse’ imbalance of sequencing reads aligning to the −ve reference strand upstream of the binding site, and reads aligning to the +ve reference strand downstream of the binding site (Supplementary Figure S4). Although this imbalance is prominent in the data generated using the newer double-hit protocol used for all recent ENCODE DNase-seq data, the pattern is less pronounced in older data generated by the single-hit DNase-seq library preparation protocol (9) (Supplementary Figure S5).

It is also evident that more DNase I cut sites are detected immediately adjacent to the DNase I footprints, perhaps because the non-protected regions of a DHS are cleaved multiple times, with the smaller fragments being lost from the analysis. Overall, the number of reads aligning to the positive and negative strands in each DHS is roughly equal (Supplementary Figure S1) and so does not account for this imbalance. For some but not all motifs, additional information can be gained by re-orienting the DNase-seq data according to the orientation of the specific motif (Supplementary Figure S6). In the case of CTCF, a region of DNase I hypersensitivity exists on the −ve strand in a region that separates the major CTCF consensus motif from a secondary CTCF-binding site reported by others (13,22,23) When the motifs are aligned in the same orientation, this second site appears as a separate distinct protected region in Supplementary Figure S6. Here, we also show that CTCF motif scores are inversely correlated with Footprint Occupancy Scores, revealing that poorer motifs are less likely to generate clear footprints, as they are more susceptible to DNase I cleavage within the binding sites.

To assess whether the consideration of strand imbalance in DNase-seq data surrounding protein–DNA binding sites has an equally significant impact on the accuracy of DGF, we developed Wellington, a novel algorithm that performs DGF on DNase-seq data without the need for any prior knowledge, such as position weight matrices for the motifs that are likely to be annotated as a footprint. Wellington makes use of the sequence tag strand imbalance and searches DHSs for footprints that have a statistical enrichment of reads aligning to the +ve and −ve reference strand upstream and downstream of the binding site, respectively, with a depletion of reads on both strands in the region of the binding site. Figure 1 shows an example of such a footprint at a binding site for the TF CTCF containing a CTCF binding motif in the K562 data. This example demonstrates that Wellington footprints can accurately recapitulate the presence of a bound protein at a known TFBS.

**Figure 3.**
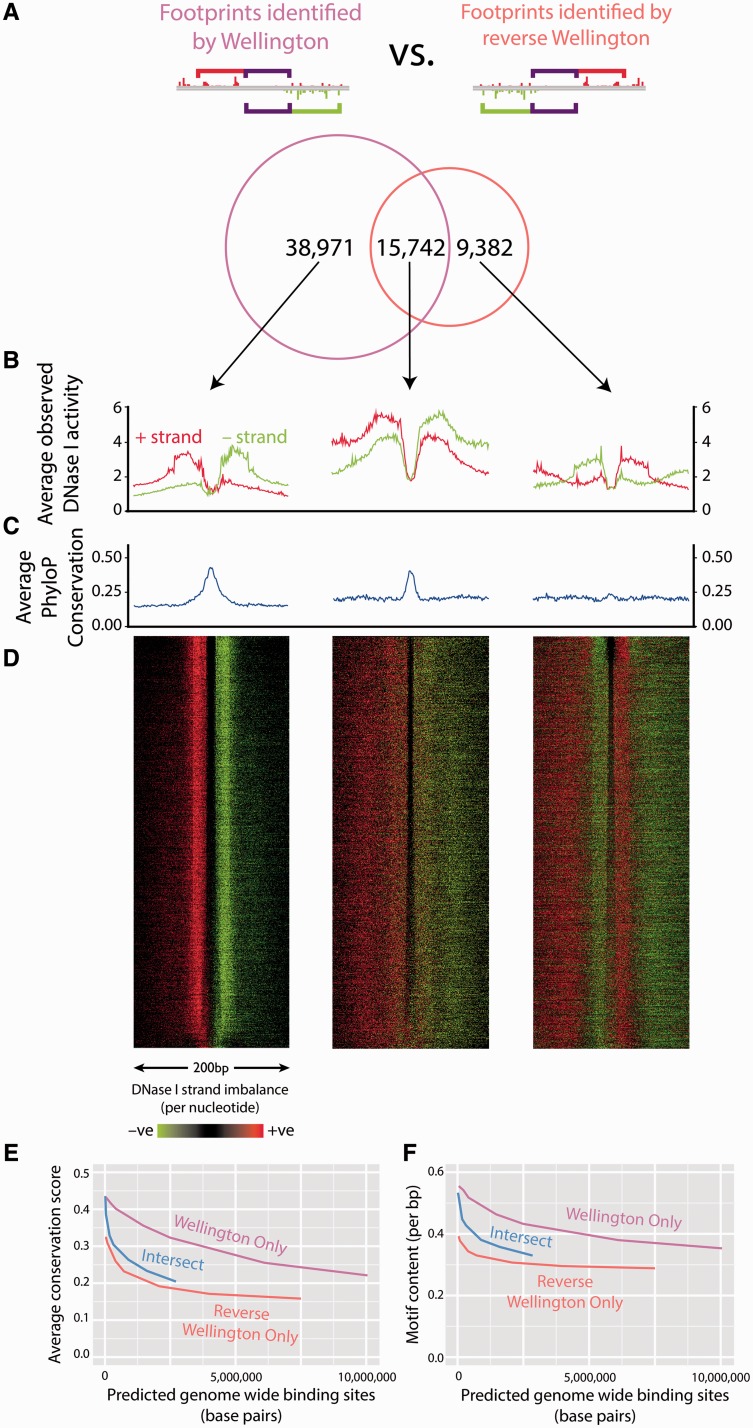
Strand imbalance information is crucial for the identification of true protein–DNA interactions. (**A**) The Wellington algorithm was run on K562 DNase-seq data in parallel with a modified version of the Wellington algorithm (reverse Wellington) designed to identify strand imbalance in the opposite direction than expected, i.e. reads aligning to the negative reference strand upstream of the binding site, and reads aligning to the positive reference strand downstream of the binding site. (**B**, **C**) Although footprints identified only by reverse Wellington harbour the characteristic depletion of DNase I cleavage, we find that they do not exhibit the increase of conservation typical of known protein–DNA interactions (7,12–14). (**D**) Heat maps of the DNase I signal surrounding the reverse Wellington footprints support the hypothesis that false-positive footprint signals primarily arise from junctions in between adjacent protein–DNA binding sites. (**E**) The observation of low conservation scores of footprints detected by reverse Wellington is maintained when comparing Wellington and reverse Wellington footprints at a range of significance levels. (**F**) Footprints detected by reverse Wellington contain fewer TF-binding motifs.

To ensure that we were not missing genuine protein–DNA binding sites by excluding footprints that exhibited strand imbalance in the opposite direction, we again applied the Wellington algorithm to the ENCODE K562 DNase-seq data, but simultaneously applied it in a ‘reverse’ mode. This detected features exhibiting strand imbalance in the opposite direction to that which we demonstrated in Figure 2, (i.e. reads aligning to the −ve reference strand upstream of the binding site, and reads aligning to the +ve reference strand downstream of the binding site). Using the reverse Wellington method, we made footprinting predictions and compared them with those made by Wellington at the same *p*-value threshold of 1 × 10^−^^30^ (Figure 3A). All footprints identified possess the typical depletion in DNase I signal at the centre of the footprint (Figure 3B and D). As it is known that sequence conservation is correlated with the strength of TF binding (5,7,12–14), we investigated PhyloP (24) conservation scores surrounding footprints identified by both Wellington and reverse Wellington. We discovered that footprints only identified by Wellington showed an enrichment in sequence conservation at the centre of the footprint. This also held true for the footprints identified by both algorithms (due to there being sufficient reads on both strands for both methods to detect a footprint). However, ‘reverse footprints’ identified by reverse Wellington only, did not show any evidence of enrichment in conservation score (Figure 3C), suggesting they are artefacts. To exclude the possibility that this result was only associated with the specific significance threshold chosen, we ran this analysis over a range of significance thresholds, but the main outcome of the analysis did not change (Figure 3E). Another indicator of the quality of footprint predictions, motif content (7,12–14), was also investigated. We found that motifs were enriched at the centre of footprint predictions (Supplementary Figure S7) and that over a range of significance thresholds, the pattern in the average motif content was the same as the average conservation score, with Wellington outperforming reverse Wellington (Figure 3F). Based on the fact that ‘reverse footprints’ with reverse strand imbalance patterns had very low motif content and very low average conservation scores, we consider these to be largely false positives. The majority of these are found adjacent to (5041, 54%), or in between (2734, 29%) footprints identified by Wellington (Supplementary Figure S8), with the minority (1607, 17%) having no neighbouring footprint within 50 bp. This indicates that these false positives are ‘ghost’ sites identified between or next to the shoulder regions of true footprints. To a strand-agnostic algorithm, these will appear to be depletions in DNase I activity associated with protein–DNA binding events. It is only by considering the strand information that it becomes possible to identify and discard them as artefacts in the data.

**Figure 4.**
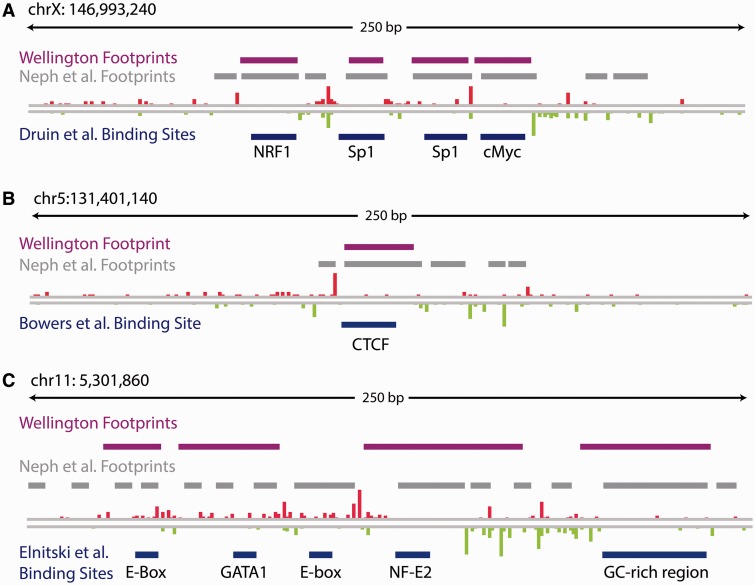
Wellington footprints recapitulate known protein–DNA interactions at (**A**) the *FMR1* promoter (25), (**B**) the *IL3* +4.9 kb insulator (26) and (**C**) the β-globin HS2 hypersensitive site (27) and refine previous footprinting predictions at these loci (7).

We next visualised footprints identified by Wellington at regions with known protein–DNA interactions that have previously been characterised by manual footprinting approaches, including the *FMR1* promoter (25), the IL-3 gene +4.9 kb CTCF site (26) and the β-globin LCR HS2 DHS (27). Figure 4 and Supplementary Figure S7 demonstrate the high precision with which Wellington infers regions of protein–DNA interaction.

### Wellington is highly accurate at inferring protein–DNA interactions from DNase-seq data

**Figure 5.**
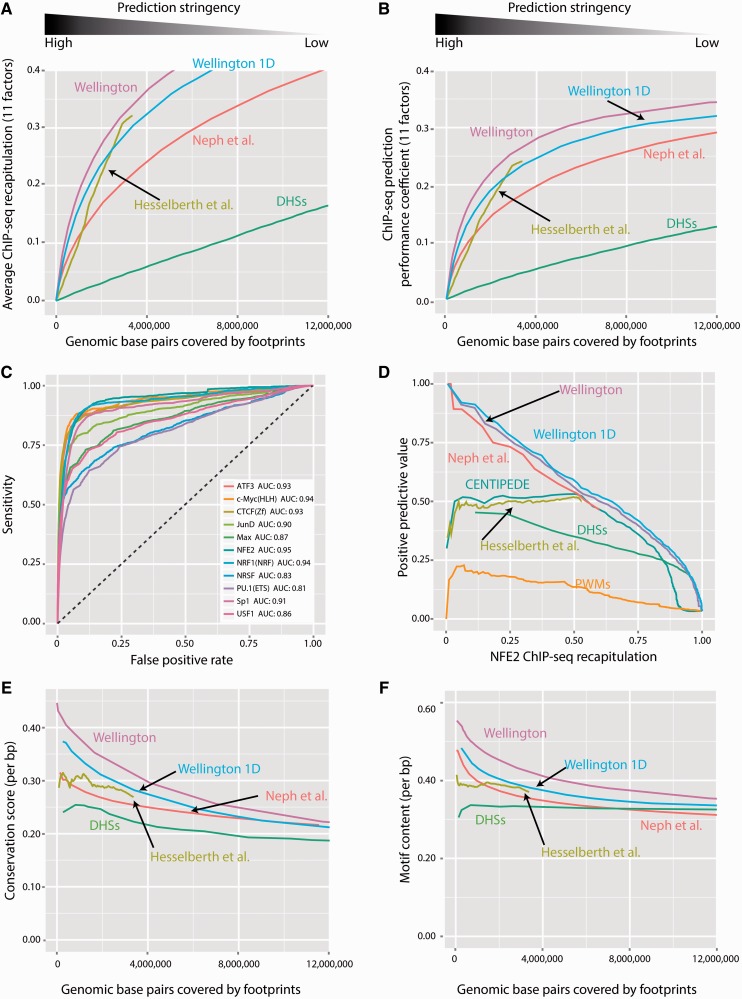
Wellington outperforms other methods with respect to ChIP-seq recapitulation performance, sequence conservation and motif content within footprints. (**A**) Wellington is able to recapitulate a given amount of ChIP-seq data with approximately half the number of genomic predictions compared with Neph *et al.* (7). The horizontal axis shows the total number of base pairs in the genome that are covered by footprints at a given stringency, the vertical axis shows the average performance of these footprints in recapitulating binding sites found from ChIP-seq data for 11 TFs in K562 cells. DHSs: using DNase hypersensitive sites to recapitulate ChIP-seq binding sites. (**B**) The nucleotide performance coefficients for these predictions (28) take numbers of false positives and false negatives into account and show a consistent finding compared to (A). (**C**) ROC curves for Wellington binding site predictions of 11 genomic TFs. The dashed line shows the expected performance of a random classifier. AUC: Area under curve. (**D**) Using the NFE2 ChIP-seq data as an example, we illustrate that the positive predictive value (the proportion of binding site predictions that are correct) of Wellington is either equal to or exceeding other footprinting techniques. (**E**, **F**) Wellington footprints have consistently the highest PhyloP conservation scores and motif content.

To further assess the performance of the Wellington algorithm at identifying protein–DNA interactions compared with other methods, we used a range of different validation techniques, again using DNase-seq, footprinting and ChIP-seq data published by ENCODE. We also considered an implementation of Wellington that ignores strand information in the data, ‘Wellington 1D’ (see supplementary material for details), to assess the impact of the strand information on footprinting performance independently of the footprinting method. In the first instance, we compared our footprinting predictions for the K562 DNase-seq data with K562 ChIP-seq data for a range of TFs (ATF3, c-Myc, CTCF, JunD, Max, NFE2, NRF1, NRSF, PU.1, Sp1 and USF1). We investigated the ChIP-seq recapitulation performance of our method by searching for motifs within footprints using a range of decreasing stringencies for the footprint *p*-value (Figure 5A). Over all stringencies, Wellington performed the best, meaning that the efficiency of Wellington at recapitulating ChIP-seq data per base pair of prediction was higher than that of other methods. For example, it required approximately 60% fewer predictions compared with Neph *et al.*’s footprint analysis to recapitulate an equal amount of ChIP-seq data for these 11 TFs. Although this analysis clearly showed the increased coverage gained by Wellington, it did not take the number of false positives or false negatives made by these predictions into account. To address this, we calculated the Average Nucleotide Performance Coefficients (28) for the 11 ChIP-seq experiments as a function of total genomic footprint predictions, which revealed a consistently higher correlation between the ChIP-seq confirmed binding sites and the Wellington footprints across all sensitivities compared with other methods (Figure 5B).

A validation method commonly used in classification experiments, the Receiver Operator Characteristic (ROC), assesses the performance of a binary classifier over a range of significance thresholds (see supplemental material for details). Wellington yielded an area under the ROC curve higher than 0.80 in the ability to recapitulate all 11 TFs in K562 cells (Figure 5C), indicating that Wellington is an excellent predictor of TF binding (29). ROC analysis was also performed on HepG2 and A549 DNase-seq data (Supplementary Figures S10 and S11), yielding similar performance. Although this method has been used in the validation of previous footprinting methods, it should be noted that due to the relatively small number of true positives (bound motif instances) and large number of true negatives (unbound motif instances) in the genome for most TFs (Supplementary Table S1), this statistic is skewed towards assessing the ability of an algorithm to correctly predict unbound locations.

CENTIPEDE (14) is based on known binding motif locations and learns one footprint model for each individual motif. It is therefore capable of using features of footprints that are specific to one or few motifs. In contrast, Wellington is a generic footprinting method for the detection of a wide range of binding sites. It does not depend on previous knowledge of motifs and does not learn models for individual motifs. We therefore considered the possibility that CENTIPEDE might outperform Wellington. However, we found that Wellington still outperformed CENTIPEDE when comparing the Positive Predictive Value (the fraction of predicted binding sites that are confirmed in ChIP-seq data, PPV) as a function of the ChIP-seq coverage (Figure 5D), implying that Wellington can be specifically used for the purpose of determining *in vivo* occupancy of a given motif. The method by Neph *et al.* and Wellington performed comparably when the location of a binding motif was known, but CENTIPEDE’s Positive Predictive Value was lower at lower sensitivities. Comparable results were observed for the other 10 TFs (Supplementary Figure S9). However, it is worth noting that when performing analyses that require the presence of a motif in the footprint, a high number of motif-less footprints are masked and unknown motifs are not found. Moreover, the assumption that a given TF generates a uniform digestion pattern limits the predictive power of the algorithm, for example, it has been shown that multiple clusters of DNase I cleavage patterns exist for CTCF (13). In addition, the dynamic binding behaviour of a specific TF can be modulated by interaction with other factors binding within the DHS (30). The extent of this has not yet been investigated, and other TFs could also generate differing DNase I cleavage patterns dependent on differing binding dynamics at individual sites across the genome.

All of the aforementioned analyses rely on ChIP-seq data as a gold standard, and therefore false positives in ChIP-seq analyses can appear as false negatives in footprinting assays and *vice versa*. Other metrics that do not rely on ChIP-seq data, such as conservation scores and motif enrichment, which are also highly correlated with TF binding and regulatory activity (31), can be used to assess footprinting performance. We therefore calculated the average PhyloP conservation score and the average motif content of footprints across a range of thresholds on footprint *p*-values. To calculate motif content, we used a library of 214 ChIP-seq derived DNA motifs. Across all sensitivities, Wellington footprints yielded higher conservation scores and motif content per base pair (Figure 5E and F) than other methods, further demonstrating Wellington’s ability to identify footprints enriched for regulatory elements with high conservation scores and protein binding potential. This notion is exemplified in Supplementary Figure S12, which depicts the DHS at the *FMR1* promoter demonstrating the precise overlap of regions with high footprinting *p*-values and high conservation scores. The ability for Wellington to outperform Wellington 1D in these metrics confirms that the consideration of the strand information in DNase-seq data assists in reducing the number of low conservation scoring false-positive ‘reverse’ footprints in the genome without affecting predictive power. When considering data generated with the original single-hit protocol, however, we found that Wellington did not improve over Wellington 1D (Supplementary Figures S14–S16). This is likely due to the fact that the single-hit data have less pronounced strand imbalance patterns (Supplementary Figure S5), which Wellington is specifically designed to detect.

In summary, Wellington efficiently increases the specificity of footprint detection by avoiding artefacts, which only become apparent when considering the alignment strand of DNase I cuts in DNase-seq data (Supplementary Figure S13). It therefore maintains excellent ChIP-seq recapitulation performance whilst significantly reducing the total number of predicted footprints in the genome.

### pyDNase: a Python package for analysing DNase-seq data

**Figure 6.**
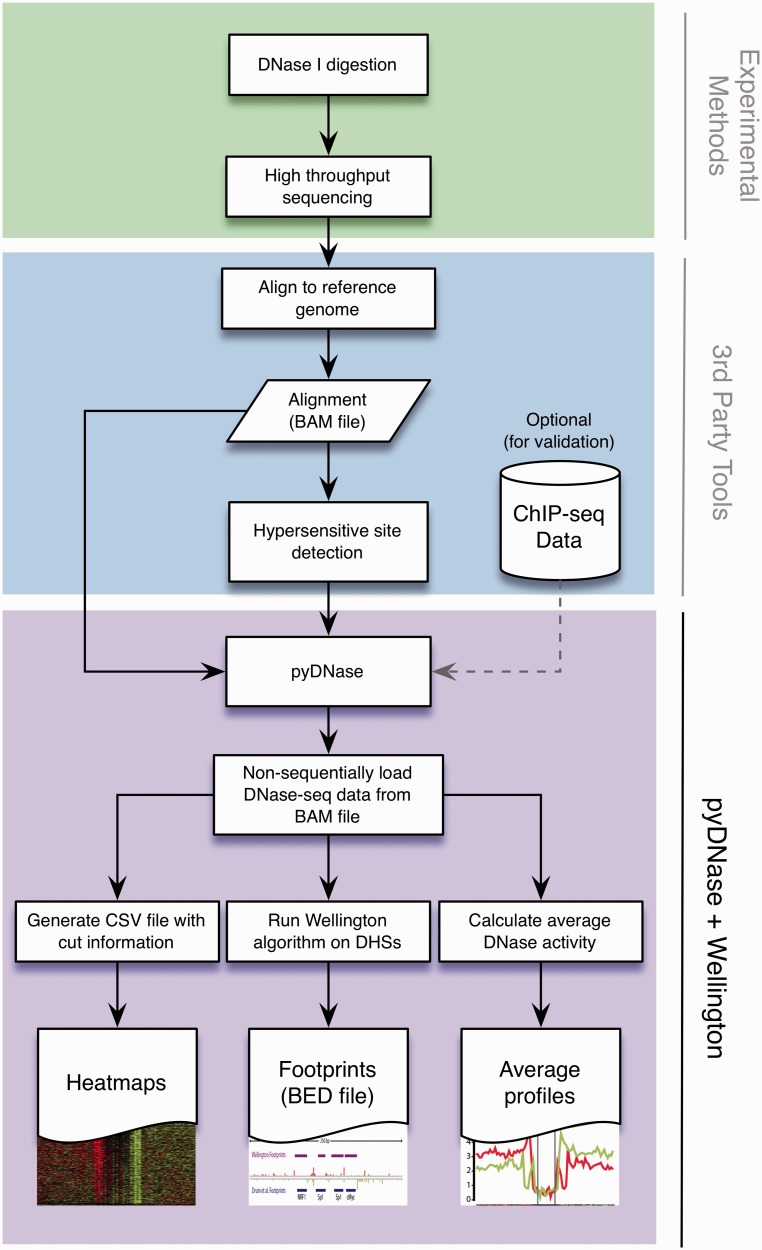
The pyDNase Python package forms a complete toolchain for the rapid analysis and footprinting of DNase-seq data. Using mapped DNase-seq reads as a BAM file, pyDNase not only has scripts to perform common analyses (heat maps, footprinting, average profiles) but also exposes an API to allow the easy development of further DNase-seq analysis tools.

At present, no free open source software package is available that would allow the analysis of DNase-seq data with the aim of performing digital footprinting without specifying any prior parameters, such as motif of interest. DGF presents unique challenges in data handling due to the large (>500 million) number of reads, and the necessity to interact directly with raw alignment data to perform complex analyses. With ChIP-seq, this step is unnecessary after basic peak calling and generation of extended read densities. We therefore developed pyDNase as the first open source DNase-seq analysis software package. pyDNase complements other common bioinformatics tools to establish the first functional DNase-seq footprinting pipeline. It is written in Python for higher-level functions and C for lower-level performance-critical functions. The analysis pipeline using pyDNase is outlined in Figure 6, whereby pyDNase serves a conduit between the raw alignment data and DNase-seq analysis algorithms such as Wellington. The most basic usage, a footprinting analysis with the default parameters can be performed by running the wellington_footprints.py script with the sequencing reads in BAM format, a list of DHSs in the data set, and an output location for the results (e.g. $ python Footprint.py reads.bam dhs.bed ∼/results/), which will then output the footprint scores as a wig file, and footprints at various *p*-value cutoffs. The behaviour of this script is highly configurable through command line arguments. pyDNase allows Wellington footprinting of all DHSs in a 600 million read DNase-seq experiment in ∼4 –10 h on a desktop computer with 1 Gb of RAM and a 2.3 GHz Intel Core i5 processor. This will simplify and expedite data analyses as well as method development for future studies. pyDNase and the Wellington algorithm are available as a Python package, along with sample data sets, a step-by-step tutorial, and documentation of every method and class at http://jpiper.github.com/pyDNase and is freely released under the GNU GPLv3 open source software license.

## DISCUSSION

By designing the Wellington algorithm to identify footprints using the knowledge that strand imbalance surrounds known protein–DNA interactions, we have increased our ability to perform DGF by reducing the number of motif-depleted non-conserved false positives. Footprints identified by Wellington show consistently higher average conservation scores, motif content and ChIP-seq recapitulation per base pair than other methods. Considering that the ChIP-seq recapitulation performance was the justification behind the previous claim of 0.4 to 2.3 million genomic footprints (dependent on the cell type) (7), the results presented here suggest that much less of the genome may be involved in protein-binding events than previously predicted. Wellington required approximately 60% fewer predictions compared with Neph *et al.*’s footprint analysis to recapitulate an equal amount of ChIP-seq data for 11 TFs. This is due to the large number of motif-less false positives in the Neph *et al.* set of predictions that do not impact on the chosen validation metrics. However, it remains difficult to determine exactly how many binding sites there may actually be as human DGF is still limited by sequencing depth (7) (Supplementary Figure S2).

We hypothesise that the strand imbalance is a natural consequence of the size selection step of the ‘double-hit’ protocol, which purifies ∼50–200 base pair DNA fragments produced by DNase I digestion (Supplementary Figure S3). This is strengthened by the result that consideration of strand information does not contribute any predictive power to data generated by the single-hit DNase-seq method, which does not use size selection in the library preparation (9) and has detectable but less pronounced strand imbalance patterns (Supplementary Figures S5, S14–S16). In the double-hit protocol, eliminating the smallest digestion products and excluding larger chromatin fragments creates a bias towards sequencing DNA fragments that actually span the DNase I footprints where TFs are bound. Because the +ve and −ve strand sequence tags simply represent the opposite ends of the same sets of DNA fragments, this is a straightforward predictor of the location of a footprint relative to the 5′ end of the sequence tag. Giving due consideration to the introduction of strand imbalance surrounding sites protected by protein–DNA interactions in the double-hit DNase-seq data allows the development of analyses that reduce the number of false positives in footprint predictions.

This increased footprinting precision as well as the ability of Wellington to be used on *a priori* defined motifs opens the door to higher-order analyses, such as *de novo* identification of occupied *cis*-regulatory modules, as well as the elucidation of direct or indirect TF interaction in a given complex *via* determination of specific motif distances. Furthermore, the strand-specific cleavage patterns surrounding motifs bound by different TF families seemingly constitute unique, individual signatures, which may permit motif identification based solely on DNase-seq data.

The identification of TFBSs bound in a cell-type and cell-stage specific fashion is a key stage in gaining an understanding of differential gene expression underlying all cell differentiation processes. Using techniques such as DNase-seq, ChIP-seq, and algorithms such as Wellington, we can begin to document the TF-binding events that confer cell identity, developmental processes or which underpin aberrant regulation in diseases such as cancer. By significantly reducing the number of false-positive predictions, we decrease the need for multiple technical and biological replicates, which can be difficult to obtain for primary tissues such as patient samples. This opens up the possibility of performing analyses on disease-specific transcription regulation mechanisms, which have previously only been possible using data combined from multiple experiments over large numbers of cell lines (7,13).

It remains to be seen how footprinting algorithms can be further enhanced. Even though it is known that the pattern of the DNase-seq signal surrounding protein–DNA binding events is TF dependent, we found Wellington to perform well using a single model to search for all possible TF-binding events in a DNase-seq data set. The use of more complex mixture models could yield even better performance, which at some stage may even allow the incorporation of an analysis of the chromatin landscape. The speed at which new computational analyses of DNase-seq data are being developed is greatly surpassed by the rate at which new DNase-seq data are being generated (32). To encourage further investigations, we have released pyDNase and Wellington as a Python package for the fast and easy analysis of DNase-seq data. We hope that accelerates both the analysis of DNase-seq data and the development of advanced footprinting algorithms.

## SUPPLEMENTARY DATA

Supplementary Data are available at NAR Online, including [33].

## FUNDING

Engineering and Physical Sciences Research Council [EP/P50578X/1 PhD grant to J.P.] (in part), a Chancellor’s Scholarship from the University of Warwick and a PhD Fellowship from the German National Academic Foundation (to M.C.E); Leukaemia & Lymphoma Research (to C.B. and P.N.C.) as well as from the Biotechnology and Biological Sciences Research Council [BB/I001220/1 to C.B.]. Funding for open access charge: RCUK block funding to University of Warwick Library.

*Conflict of interest statement*. None declared.

## Supplementary Material

Supplementary Data
