# Wellington: a novel method for the accurate identification of digital genomic footprints from DNase-seq data

**DOI:** 10.1093/nar/gku727

**Published:** 2014-09-26

**Authors:** Jason Piper, Markus C. Elze, Pierre Cauchy, Peter N. Cockerill, Constanze Bonifer, Sascha Ott

Nucl. Acids Res. (2013) 41 (21): e201. doi: 10.1093/nar/gkt850

It has come to the authors’ attention that several presentation errors exist in this article that incorrectly describe the Wellington method.

The formula on page 3 that reads

${\rm p\hbox{-}value = \{1-F[FP^{+}, FP^{+} + SH^{+}, l_{FP}/(l_{FP}+l_{SH})]\} \cdot \{1-F[FP^{-}, FP^{-} + SH^{-}, l_{FP}/ (l_{FP}+l_{SH})] \ ]}$

should read as follows

${\rm p\hbox{-}value = \{F[FP^+, FP^+ + SH^+, l_{FP}/(l_{FP}+l_{SH})]\} \cdot \{F[FP^-, FP^- + SH^-, l_{FP}/ (l_{FP}+l_{SH})] \ ]}$

We also present the following update to Figure 1, as the original did not specify a logarithmic y-axis, and to clarify the figure legend.

**Figure 1.**
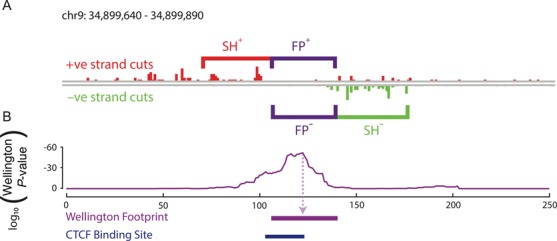
Wellington: a novel strand sensitive algorithm for the identification of protein–DNA binding sites from DNase-seq data. (A) The Wellington algorithm calculates *p*-values for every base pair in all DNase hypersensitive sites in a given DNase-seq data set, where the *p*-value is assigned to the base pair at the centre of the footprint. For each base pair, Wellington tests the hypothesis that there are significantly fewer reads aligning to the forward reference strand footprint region (FP^+^) than to the forward reference strand in the upstream shoulder region (SH^+^) and significantly fewer reads aligning to the reverse reference strand footprint region (FP^−^) than to the reverse reference strand in the downstream shoulder region (SH^−^). (B) Example output of the Wellington algorithm. The corresponding footprint prediction recapitulates the ChIP-seq confirmed CTCF-binding site.

These errors are purely typographical and do not affect the underlying methods, results or conclusions of the manuscript. We apologise for any confusion caused.

