# Supplementary material for: Wellington: a novel method for the accurate identification of digital genomic footprints from DNase-seq data
Source: Nucleic Acids Res. 2013 Sep 25;41(21):e201. doi: 10.1093/nar/gkt850 (PMC3834841; doi:10.1093/nar/gkt850)
Supplement: Supplementary Data [file supp_41_21_e201__index.html]

Wellington: a novel method for the accurate identification of digital genomic footprints from DNase-seq data — Wellington: a novel method for the accurate identification of digital genomic footprints from DNase-seq data — Supplementary Data 

# Wellington: a novel method for the accurate identification of digital genomic footprints from DNase-seq data

## Supplementary Data

files

**Files in this Data Supplement:**

- Supplementary Data - zip file
