# Supplementary material for: Wellington: a novel method for the accurate identification of digital genomic footprints from DNase-seq data
Source: Nucleic Acids Res. 2013 Sep 25;41(21):e201. doi: 10.1093/nar/gkt850 (PMC3834841; doi:10.1093/nar/gkt850)
Supplement: Supplementary Data [file supp_gkt850_suppl_data.zip › nar-01319-met-f-2013-File008.pdf]

Contents

1 Development of the Wellington Algorithm **2**

1.1 Goals and underlying assumptions . . . 2

1.2 Rationale . . . 2

1.3 Data preprocessing . . . 3

1.4 Calculating a p-value for a given potential footprint . . . 4

1.5 A strand agnostic Wellington . . . 5

1.6 Selecting footprint and shoulder widths at a given position . . . 5

1.7 Greedy selection of footprints from all possible candidates . . . 6

1.8 Choosing a significance threshold and assessing possible false positives . . . 6

1.9 Sequencing Depth . . . 7

1.10 Interpretation of the footprinting results . . . 8

1.11 Options to increase computational efficiency . . . 9

1.12 Alignability of the genome . . . 9

1.13 Possible extensions to the Wellington method . . . 10

2 pyDNase **10**

3 Validation of Footprints **11**

3.1 ChIP-seq data . . . 11

3.2 CENTIPEDE and ENCODE data preparation . . . 15

3.3 Definition of performance characteristics . . . 15

3.4 Performance statistics . . . 16

3.5 Conservation and motif content . . . 16

4 Supplemental Figures **17**

1 Development of the Wellington Algorithm

1.1 Goals and underlying assumptions

The goal of Wellington is to statistically identify footprints from DNase I cut data. We define a footprint as a region where the number of DNase I cuts per base pair is significantly lower than in the surrounding area. Specifically, we compare the number of DNase I cuts per base pair inside the possible footprint region on the forward reference strand with cuts per base pair the upstream region on the forward reference strand and the number of DNase I cuts per base pair inside the possible footprint region on the backward reference strand with cuts per base pair the downstream region on the backward reference strand.

As stated in the Methods, we assume that the number of DNase I cuts is much lower (depleted) in regions of closed chromatin or of open chromatin with a bound protein than in regions of open chromatin without a bound protein, which is well established by the literature [1, 2, 3, 4, 5]. Thus, protein-DNA binding sites can be detected by finding a characteristic depletion of DNase I cuts compared to the surrounding region of open chromatin without bound proteins. Furthermore, we assume that the number of DNase I cuts in open chromatin without bound proteins is roughly proportional to the length of the region. Thus, we can test if a region has a significantly lower than expected number of cuts to identify footprints or putative protein-DNA binding sites.

1.2 Rationale

It has been previously established that DNase I cuts are not distributed uniformly across the open chromatin, but that the probability is dependent on the DNA sequence around and at the cleavage site. This problem could theoretically be compensated by appropriate pre-processing, such as adjusting the observed number of reads by the DNase I cutting rates for the surrounding base doublet [6]. However, in practice, we and others have found that differences in cutting preferences are sufficiently small to not have an undue impact on the footprint identification. The difference in DNase I accessibility within and outside DHS in chromatin by far exceeds sequence dependent differences in the digestion frequency of naked DNA [5, 3].

Due to the methodology employed, DNase-seq typically only includes DNA fragments of a certain size range. Any fragments smaller than about 50bp or larger than about

250bp are discarded. Thus, DNase I needs to cut the DNA twice in reasonably close proximity for the fragment to be included in the analysis. This means that regions with multiple bound proteins in close proximity to each other or regions with bound proteins close to nucleosomal chromatin might be hard to identify. In order to avoid this problem as much as possible, we only consider cuts arising from fragments that span across the footprint site, i.e. those upstream of the footprint site on the forward strand and those downstream of the possible footprint site on the reverse strand. In principle, other bound proteins very close to the possible footprint site might still be a problem even with this step. However, this was not observed in practice.

The DNA fragment may be further shortened by additional DNase I cuts. This means that we typically expect to observe more shorter DNA fragments than longer DNA fragments. This can lead to an increased number of cuts just outside of footprints upstream on the forward strand and downstream on the reverse strand. Wellington currently does not explicitly utilise this phenomenon, as we did not observe it for all footprints.

1.3 Data preprocessing

Different DNase-seq techniques can produce sequencing artifacts, e.g. in the form of read spikes at single base pairs. If possible, appropriate preprocessing should be used in order to reduce the impact of sequencing artifacts and other undesirable phenomenona mentioned above. Which preprocessing method is appropriate depends on the precise experimental method. Some authors have suggested to allow a maximum number of reads per base pair to reduce the impact of spikes. Whilst the provided software implementation of the Wellington algorithm offers this feature, we do not generally recommend it, as a lot of data may be needlessly discarded.. Rather, we encourage researchers either to identify and remove sequencing artifacts manually or to choose a sufficiently high p-value cutoff and to check for problems by shuffling the data and searching for footprints again (see below). Usually, the forward and the reverse strands will have a similar number of cuts (Figure S1). Thus, finding many more cuts on one strand than on the other in a region can be an indicator that a sequencing artifact may be present.

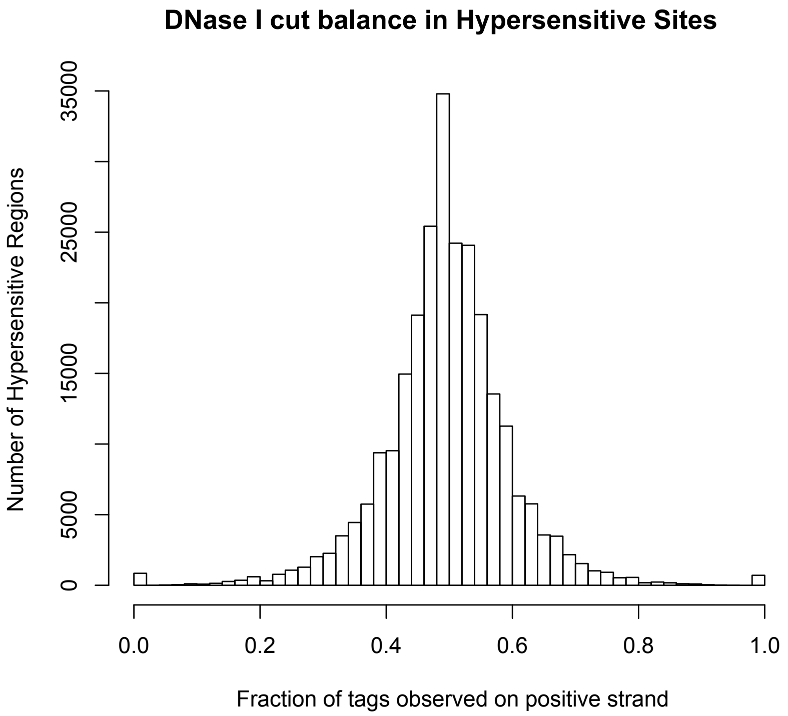

Figure S1: The numbers of cuts observed on the positive and on the negative strand are of the same order of magnitude. Within each hypersensitive region in K562 cells, the ratio of cuts on the positive strand to the total number of cuts has a mean of 0.50 and a standard deviation of 0.10.

1.4 Calculating a p-value for a given potential footprint

As described in the Methods, we use the following notation introduced in Figure 1. We call the region surrounding the possible footprint the shoulder region. Let l_{FP} be the length of the possible footprint and l_{SH} be the length of the shoulder on each side of the possible footprint. For now, consider l_{FP}, l_{SH}, and the centre of the potential footprint as given. We can then calculate the four DNase I cut counts that are relevant for the hypothesis test: the total number (i.e. sum over all base pairs) of cuts on the forward reference strand inside the possible footprint (FP^+), the number of cuts in the upstream shoulder region on the forward reference strand (SH^+), the number of cuts on the backward reference strand inside the possible footprint (FP^-), and the number of cuts in the downstream shoulder region on the backward reference strand (SH^-).

We test the null hypothesis that the number of cuts is proportional to the region length by using a binomial test. Because the number of cuts might depend on the strand, e.g.

because the protein structure might be such that it only inhibits DNase I activity on one strand, we test both strands separately. With $F(k, n, p)$ being the binomial cumulative distribution function (the probability of achieving at least k out of n successes for the probability of each success being p), we calculate a p-value using the formula

$$p - value = F(FP^+, FP^+ + SH^+, \frac{l_{FP}}{l_{FP}+l_{SH}}) * F(FP^-, FP^- + SH^-, \frac{l_{FP}}{l_{FP}+l_{SH}})$$

This p-value is for a given possible footprint of size l_{FP} with surrounding shoulder regions of size l_{SH}.

1.5 A strand agnostic Wellington

In order to investigate the impact of the strand information of Footprinting results indepently of footprinting methodolody, we utilised a simplified version of Wellington which uses data on both strands, Wellington 1D. We calculate parameters in the model differently than above to account for this. Let the total number of cuts on both strands inside the possible footprint (FP), the number of cuts in the upstream shoulder region on the both strands (SH^u), and the number of cuts in the downstream shoulder region on both strands (SH^d). We then calculate a p-value using the formula

$$p - value = F(FP, FP + SH^d + SH^u, \frac{l_{FP}}{l_{FP}+l_{SH}})$$

1.6 Selecting footprint and shoulder widths at a given position

For a given centre position of a possible footprint, we can vary both the length of the possible footprint l_{FP} and the length of the shoulder l_{SH}. This results in a multitude of hypothesis tests, all of which may have different p-values. Typically, the researcher will specify a range of possible values for l_{FP} and l_{SH} that are appropriate. If no hypothesis tests are significant at the chosen significance threshold, there is clearly no evidence for this site being a footprint. If only one hypothesis test is significant at the chosen significance threshold, we consider this a footprint of length l_{FP} belonging to the significant test. Matters get slightly more complicated if more than one test is significant.

If more than one test is significant, we have successfully rejected multiple slightly different hypotheses. For practical purposes, we wish to have a set footprint length l_{FP} and shoulder length l_{SH} instead of multiple possible values. To achieve this, we choose the l_{FP} and l_{SH} that provide the most evidence against the null hypothesis and result in the lowest p-value. From a Bayesian perspective, this corresponds to putting a uniform

prior over the previously specified ranges of possible l_{FP} and l_{SH}. It is straightforward to extend Wellington to allow arbitrary priors for l_{FP} and l_{SH}.

1.7 Greedy selection of footprints from all possible candidates

The previous section produced one p-value for each possible footprint centre base pair along with corresponding footprint widths. If we only have one significant p-value in a region, the corresponding possible footprint will be considered our one true footprint. Multiple significant p-values in a region may result in overlapping footprints and a decision has to be made how to deal with this phenomenon.

While overlapping footprints can occur and we wish to allow this, we also want to avoid artificially extending footprints simply for the reason that base pairs slightly away from the centre of a protein binding site will often still succeed in rejecting the null hypothesis. Thus, we wish to require two overlapping footprints to overlap for less than a certain user-settable percentage, which defaults to 50%. This requirement in no way restricts what footprint patterns are possible. Lifting this requirement results in noticeably larger footprints, which are often somewhat longer than the desired maximum length for a single footprint.

To achieve this goal, we implement a greedy selection strategy. For a given region, we start by choosing the footprint with the lowest p-value as our first footprint, as this footprint offers the strongest evidence against the null hypothesis. After we have added this footprint to our list of identified footprints, we then consider any base pairs contained in this footprint not to be eligible to be the centre of additional footprints. For the next footprint, we continue in the same fashion by choosing the footprint with the lowest p-value, adding it to our list, and removing all base pairs contained in it from the list of possible footprint centres. This process continues until no eligible base pairs remain with a p-value below the significance threshold.

1.8 Choosing a significance threshold and assessing possible false positives

To choose a significance threshold, the fact that possibly billions of hypothesis tests are performed needs to be considered. We decide to err on the side of caution and perform a

Bonferroni correction. To make the multiple testing correction as simple as possible for the end user, we adjust all p-values instead of just adjusting the significance threshold internally. More advanced methods, such as a Bonferroni-Holm correction, are not used for the sake of computational simplicity.

As, even with excellent preprocessing, the cut counts in open chromatin regions without bound proteins will be neither uniformly nor independently distributed, we typically recommend being more conservative than the standard $p < 0.05$ threshold (corresponding to 1.3 on the $-\log$ scale). For ENCODE datasets, we found that thresholds of $1.3 - 20$ work very well, depending on the desired number of false positives.

Ultimately, when applying this method to a dataset, we wish to adjust the p-value threshold we choose for calling footprints on a hypersensitive site-wise basis to generate a single set of footprints for the dataset (and not set a single p-value cutoff for the entire dataset). In order to do this, Wellington has a command line argument to employ an empirical method of estimating the False Discovery Rate (FDR) as described previously [5, 3]. Briefly, we shuffle the number of tags aligned to each base pair within a hypersensitive site and recalculate the footprint scores on this shuffled data 500 times, and then can determine a p-value threshold which would only occur at most 1 in 100 times, corresponding to an FDR of 0.01.

1.9 Sequencing Depth

The number of footprints called will largely be determined by the sequencing depth of the dataset. A common question from experimentalists remains 'How deep do I need to sequence my DNase-seq samples to perform digital footprinting?'. In order to assess the affect of sequencing depth on footprint detection, using the ENCODE SkMC DNase-seq dataset (owing to its large 550 million read depth), we randomly subsampled reads to simulate the effect of differing read depths. We then ran Wellington on these data, utilising an FDR of 0.01 to select footprints at the varying read depths. In line with the subsampling efforts performed by ENCODE [5]; footprint detection of human DNase-seq data by these methods are currently limited by the number of sequencing reads, with a positive correlation between sequencing depth and number of footprints detected (Figure S2). The current answer to this question, is therefore 'as much as possible', as we have not yet reached a point where contributing more sequencing depth does not increase the

number of footprints detected.

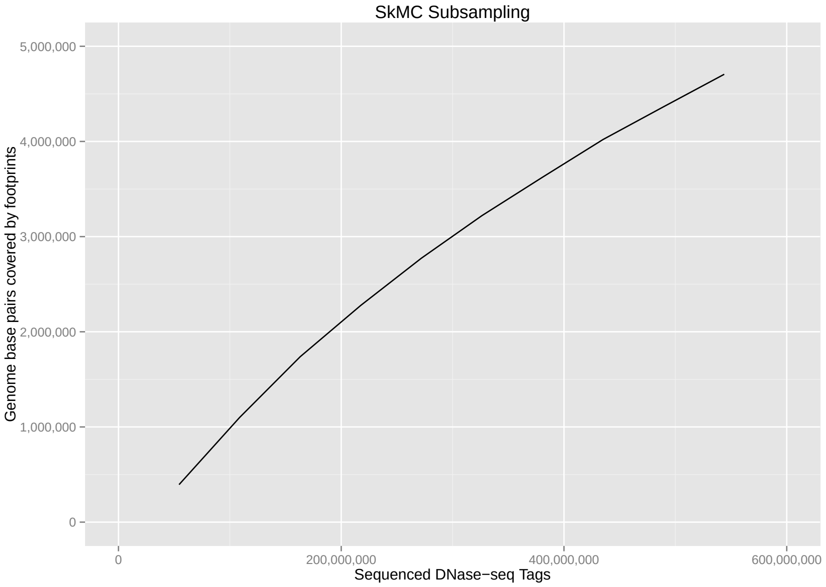

Figure S2: The number of footprints identified by Wellington in the SkMC DNase-seq dataset is positively correlated with sequencing depth. DNase-seq data were randomly subsampled in order to simulate different read depths of a single DNase-seq library, and the Wellington algorithm was used to identify footprints (FDR: 0.01).

1.10 Interpretation of the footprinting results

As mentioned before, note that p-values only give an indication of the strength of evidence against the null hypothesis, but do not provide a measure of footprint quality or strength. Further note that footprinting results depend on both the choice of significance threshold and possible values for l_{FP} and l_{SH}. We recommend trying Wellington with different parameters and observing how the choices influence the footprinting results as, in some situations, changing these can cause individual footprints to change their length or move by several base pairs. Therefore, when interpreting the footprinting results we need to be mindful of not to over-interpret minor differences in the p-values. In particular, when comparing footprints to known motifs, we recommend not requiring a 100% overlap between footprints and motifs, but some tolerance. As always, sensitivity analyses should be performed to see how any inferences made from the result depend on choices made.

1.11 Options to increase computational efficiency

There are several options to reduce computation time. The biggest gains can be achieved by restricting the search for footprints to regions of interest, such as DNase I hypersensitive sites (DHS). It has been established in the literature that the vast majority of protein binding sites are in these regions [4]. The pyDNase package can incorporate DHS coordinate information and restrict the footprint search to these regions without needing to load data from other parts of the genome. We have compared the footprinting results for ENCODE datasets for the whole genome and for DHS only and the differences in the results were small (data not shown). cl Computation time can also be reduced by reducing the number of possible values for l_{FP} and l_{SH}. For ENCODE datasets, we have found that restricting these to multiples of two does not significantly change the results.

The pyDNase package offer additional options which change how the algorithm processes data to accommodate certain computer setups (e.g. low amounts of RAM), but does not alter the algorithm nor impact the results. These are explained in the documentation provided with the package.

1.12 Alignability of the genome

We note that previous methods have included the consideration of the alignability of the target genome in their model [3], whilst others do not [7, 8]. In particular, a short unalignable region in between two alignable regions might be falsely identified as a footprint if the mappability of the genome is not taken into consideration. We initially allowed for mappability correction using a previous method [3] but the results did not yield an increase in performance and increased compute time by a factor of 5-10x, extending analysis times to several days. The negligible difference in performance was because this hypothetical situation of short, unalignable regions in the genome is not particularly common, especially as read length increases beyond 36bp. Based on this, we decided in our final model that mappability would not be considered. We recommend that anyone wishing to perform mappability correction filters footprints that are in unmappable regions using their criterion of choice after footprint detection. This method has been utilized previously [5], who note that less than 1% of their footprints satisfy this criterion.

1.13 Possible extensions to the Wellington method

The Wellington method can be easily extended in several directions. The current binomial hypothesis test can be changed to a more complex null hypothesis that takes sequencing artifacts (spikes) in the data into account. Priors on footprint lengths or even fully Bayesian approaches can easily be implemented by simply changing how the p-values are calculated. Additional preprocessing methods can also easily be added, e.g. to allow taking DNase I cutting preferences into account.

It remains to be seen how digital genomic footprinting can be used to compare multiple datasets covering differing states (e.g. healthy vs. diseased). The presence of a DHS in two cell types does not mean that the same event is occurring, as different transcription factors could be binding in a context sensitive manner. More detailed analyses into the differences in DNase cuts in DHSs between datasets would give an insight into the occupancy of promoters and enhancers by different transcription factors in differing states.

2 pyDNase

We noticed the need for a simple tool to handle DNase-seq data from a standard format, and to be able to access the data in a random order, as 90% of the genome is not hypersensitive and therefore processing these regions increases computational time and resources tenfold. pyDNase solves these problems by providing a simple Python interface to access cut information stored in the Binary Standard Alignment/Map (BAM) file format produced by popular mapping tools such as Bowtie and BWA and adopted by the ENCODE consortium as the preferred alignment file format.

pyDNase capitalises on the recent introduction of the SAM format to randomly access cut information in any region in the genome without the need to load the entire dataset at once. By using peak detection software such as FindPeaks, HOMER, Maq, HotSpots, etc to locate DNase Hypersensitive sites, footprinting algorithms can be performed solely on regions of interest, speeding up computational time dramatically.

Briefly, pyDNase uses a key-value array read cache which can be enabled or disabled at run time. If enabled, when DNase-seq cut data are requested from a genomic location for the first time, the surrounding 1000bp (configurable) will be automatically stored in memory for subsequent access. A key-value array is used as a sparse vector to store

this data, as most sequences in the genome are not hypersensitive (and therefore have a data value of 0) so the use of spare data storage significantly reduces the memory footprint. Around 4GB of RAM is required to cache all the information in Human DNase I hypersensitive sites at once.

Full documentation and description of the features can be found at

`http://jpiper.github.com/pyDNase`

3 Validation of Footprints

3.1 ChIP-seq data

We used 'optimal' ChIP-seq peaks downloaded directly from the EBI ENCODE analysis FTP server. Names of the files along with summary statistics for each ChIP-seq experiment can be found in Table S1.

Cell Type	Track Name	ChIP factor	ChIP-Seq Peaks Total	With Motif	Without Motif	Genomic Motifs Inside Peaks	Outside Peaks
K562	EncodeHaibTfbsK562Atf3V0416101	ATF3	16,011	2,062	13,849	4,298	160,476
K562	EncodeSydhTfbsK562CmycStd	cMyc	5,023	2,098	2,925	4,311	509,474
K562	EncodeOpenChromChipK562Ctcf	CTCF	56,058	25,788	30,270	26,432	41,171
K562	EncodeUchicagoTfbsK562EjundControl	JunD	26,674	2,600	24,074	5,070	112,080
K562	EncodeHaibTfbsK562MaxV0416102	Max	46,171	16,419	29,752	34,226	1,131,669
K562	EncodeSydhTfbsK562Nfe2Std	NFE2	2,637	1,619	1,018	1,750	50,360
K562	EncodeSydhTfbsK562Nrf1Iggrab	NRF1	4,211	2,609	1,602	5,960	20,440
K562	EncodeHaibTfbsK562NrsfV0416102	NRSF	15,849	2,055	13,794	2,112	2,750
K562	EncodeHaibTfbsK562Pu1Pcr1x	PU.1	28,677	18,514	10,163	20,262	549,330
K562	EncodeHaibTfbsK562Sp1Pcr1x	Sp1	7,206	2,830	4,376	4,861	137,047
K562	EncodeHaibTfbsK562Usf1V0416101	USF1	18,521	12,431	6,090	23,808	524,900
A549	EncodeHaibTfbsA549Atf3V0422111Etoh02	ATF3	6,580	308	6,272	636	164,138
A549	EncodeSydhTfbsA549Bhlhe40Iggrab	bHLHE40	3,123	1,225	1,898	2,667	254,108
A549	EncodeSydhTfbsA549CebpbIggrab	CEBP	38,845	25,305	13,540	46,517	1,722,853
A549	EncodeUwTfbsA549CtcfStd	CTCF	45,732	23,536	22,196	24,289	43,314
A549	EncodeHaibTfbsA549Elf1V0422111Etoh02	ELF1	8,611	5,075	3,536	6,937	348,645
A549	EncodeHaibTfbsA549Ets1V0422111Etoh02	ETS1	5,525	2,564	2,961	3,466	1,145,432
A549	EncodeHaibTfbsA549GabpV0422111Etoh02	GABP	12,348	7,196	5,152	9,396	871,724
A549	EncodeSydhTfbsA549MaxIggrab	Max	9,881	3,982	5,899	8,965	1,156,930

A549	EncodeHaibTfbsA549NrsfV0422111Etoh02	NRSF	11,970	1,938	10,032	1,861	3,001
A549	EncodeHaibTfbsA549Usf1V0422111Etoh02	USF1	8,004	4,710	3,294	9,452	539,256
A549	EncodeHaibTfbsA549Yy1cV0422111Etoh02	YY1	10,259	2,148	8,111	2,079	52,874
A549	EncodeHaibTfbsA549Zbtb33V0422111Etoh02	ZBTB33	7,152	626	6,526	1,052	14,443
HepG2	EncodeHaibTfbsHepg2Atf3V0416101	ATF3	3,291	1,132	2,159	2,392	162,382
HepG2	EncodeOpenChromChipHepg2Cmyc	c-Myc	4,413	1,762	2,651	3,558	510,247
HepG2	EncodeHaibTfbsHepg2Ctcfsc5916V0416101	CTCF	55,778	26,856	28,922	27,655	39,948
HepG2	EncodeHaibTfbsHepg2Foxa2sc6554V0416101	FOXA1	40,989	29,356	11,633	76,105	6,363,320
HepG2	EncodeHaibTfbsHepg2Hnf4asc8987V0416101	HNF4a	20,805	10,913	9,892	12,889	519,231
HepG2	EncodeHaibTfbsHepg2JundPcr1x	JunD	21,614	866	20,748	1,632	115,518
HepG2	EncodeSydhTfbsHepg2MaxIggrab	Max	11,854	4,707	7,147	10,726	1,155,169
HepG2	EncodeHaibTfbsHepg2Mybl2sc81192V0422111	MYB	17,898	8,016	9,882	10,306	2,389,517
HepG2	EncodeSydhTfbsHepg2Nrf1Iggrab	NRF1	1,902	1,635	267	4,132	22,268
HepG2	EncodeHaibTfbsHepg2NrsfV0416101	NRSF	12,828	1,686	11,142	1,743	3,119
HepG2	EncodeHaibTfbsHepg2RxraPcr1x	RXR	17,063	6,976	10,087	9,044	1,265,857
HepG2	EncodeHaibTfbsHepg2Sp1Pcr1x	Sp1	25,477	3,599	21,878	6,087	135,821
HepG2	EncodeSydhTfbsHepg2Srebp1InslnStd	Srebp1a	2,585	293	2,292	307	327,404
HepG2	EncodeSydhTfbsHepg2TbpIggrab	TBP	13,806	2,490	11,316	3,798	3,136,789
HepG2	EncodeSydhTfbsHepg2Tr4Ucd	TR4	2,953	660	2,293	836	88,253
HepG2	EncodeHaibTfbsHepg2Usf1Pcr1x	USF1	21,890	14,809	7,081	27,503	521,205

Table S1: Summary information of ChIP-seq data used in this study, along with statistics displaying the number of ChIP-seq peaks with and without the transcription factor's corresponding motif, and the number of the genomic motif occurrences that are within ChIP-seq peaks.

3.2 CENTIPEDE and ENCODE data preparation

All motif instances in the genome were located using HOMER, and DNase-seq cuts were exported into the custom data format required by CENTIPEDE using pyDNase. We verified that our implementation was working using the example data and results provided by the authors. CENTIPEDE was then run on each transcription factor. ENCODE [5] footprinting data were downloaded from the EBI ENCODE analysis FTP server, and sorted according to the score assigned to each footprint. Their footprints were extended 7bp in each direction as per their processing instructions. We found that when this step is ommitted, the ENCODE [5] footprints are often too small to overlap motif instances and the performance drops drastically.

3.3 Definition of performance characteristics

In order to calculate the Receiver Operator Characteristic (ROC), we must define a gold standard set of bound and unbound motifs using each ChIP-seq experiment. Using ChIP-seq derived matrices provided as part of the HOMER suite [9], we searched the entire genome for known binding motif instances. Motifs which were found inside ChIP-seq peaks were said to be bound by its corresponding factor family, and motifs falling outside the ChIP-peaks, were considered to be unbound by its corresponding factor.

We then calculated the footprint predictions over a range of p-value thresholds for Wellington, the full range (0 to 0.95) of Footprint occupancy scores for ENCODE [5], and full range (0 to 1) Log-odds probabilities for CENTIPEDE, limiting our analyses to the DHSs provided by ENCODE. Using the same set of motif locations outlined above, we then used the Wellington footprints to split all genomic instances of the motifs into 'Predicted to be Bound' or 'Not Predicted to be Bound', if either 70% of the motif was contained within the footprint, or vice versa. Thus, we end up with the following classifications for ROC analysis.

- True Positives (TPs): Motif instances falling within ChIP-seq peaks that are correctly predicted as being bound by Wellington.
- True Negatives (TNs): Motif instances falling outside of ChIP-seq peaks that are correctly predicted as being unbound by Wellington.

- False Positives (FPs): Motif instances falling outside of ChIP-seq peaks that are incorrectly predicted as being bound by Wellington.
- False Negatives (FNs): Motif instances falling within ChIP-seq peaks that are incorrectly predicted as being unbound by Wellington.

This can either be measured on a site-wise basis, or base pair basis. In practice, we found little difference between the statistics in the results presented, but used the per base pair prediction statistic.

3.4 Performance statistics

Performance statistics were calculated as follows.

- Positive Predictive Value (PPV): $TP/(TP + FP)$
- Sensitivity (Coverage): $TP/(TP + FN)$
- False Positive Rate (FPR): $FP/(FP + TN)$
- Performance Coefficient [10] (PPC): $TP/(TP + FN + FP)$

3.5 Conservation and motif content

Conservation was calculated by summing the Vertebrate phyloP46way values for each co-ordinate in a set of footprints, and then dividing by the number of basepairs to yield the average conservation per bp. Motif content was calculated by searching for motifs (using HOMER's ChIP-seq derived matrices) and then counting the number of basepairs in the predicted footprints that are overlapped by a motif (multiple overlapping motifs at one base pair do not increase the score), and then dividing by the number of basepairs to yield the average motifs per bp. *de novo* motif searching was performed using HOMER [9].

4 Supplemental Figures

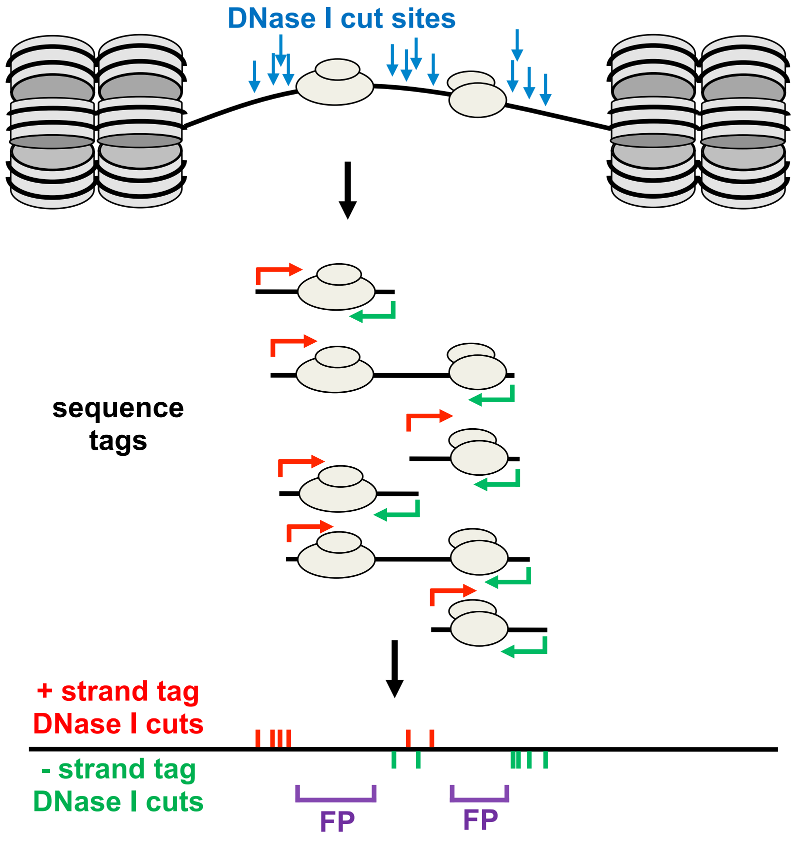

Figure S3: Chromatin structure based modeling of strand-specific DNase-seq data arising from DHSs. DHSs are usually 200-250 bp across, and the DNA sub-fragments of DHSs detected by DNase-seq are typically in the order of 50 to 150 bp in length and are surrounded by nucleosomal DNA. As depicted above, most of these fragments are expected to originate from within the DHS, meaning that they are likely span the regions of DNA protected by bound factors (indicated as ovals) that give rise to DNase I footprints (FPs). This means that it is the cut site that must be used to identify FPs, and not the entire sequence tag as is used in most peak detection algorithms. Furthermore, because sequence tags represent just one end of these fragments, upper strand +ve sequence data (red arrows) should represent sequences starting upstream of these FPs, while lower strand –ve sequence data (green arrows) should represent sequences starting downstream of FPs. The Wellington program has taken advantage of the fact that this strand information can be used to greatly increase the power of FP detection algorithms by making use of both the precise position of the cut site, and the predicted orientation of these cuts relative to a bound factor. As represented below the model, when the sum of the DNase I cuts in a DHS is depicted it is immediately apparent that FPs will generate a concentration of upstream +ve strand tags and a concentration of –ve strand downstream tags.

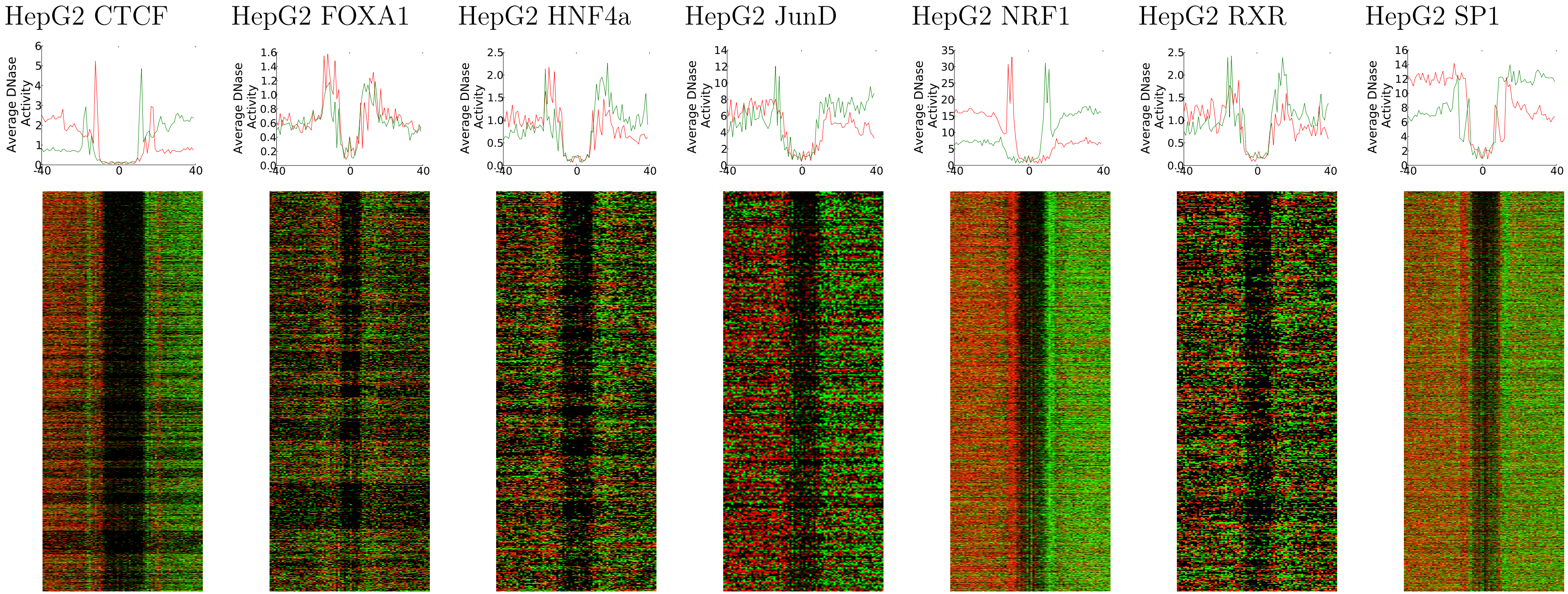
HepG2 CTCF
HepG2 FOXA1
HepG2 HNF4a
HepG2 JunD
HepG2 NRF1
HepG2 RXR
HepG2 SP1
Average DNase Activity
-40
0
40

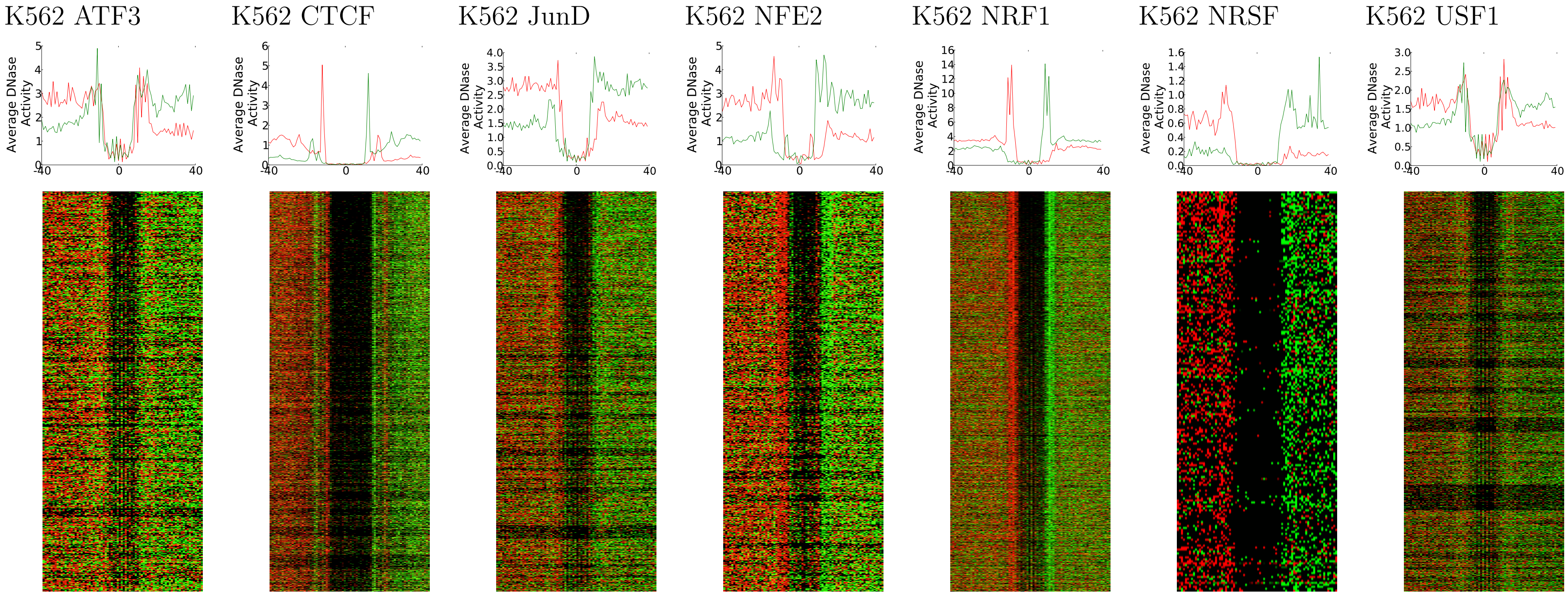

Figure S4: DNase-seq cleavage imbalance in double-hit DNase-seq data is observed at multiple transcription factor binding sites and multiple cell types. Upper panels: DNase-seq cleavage patterns surrounding ChIP-seq verified binding sites in HepG2 and K562 cells with a Footprint Occupancy Score (FOS) [5] of <0.95 illustrate the abundance of sequencing fragments aligning to the positive reference strand (red) upstream of protein-DNA binding sites, and to the negative reference strand (green) downstream of the protein-DNA binding site. Note cell-type independent differences and similarities between cleavage patterns for each transcription factor family. Lower panels: Heat map depicting cutting frequencies around the binding site. Binding sites are sorted from top to bottom in order of decreasing FOS .

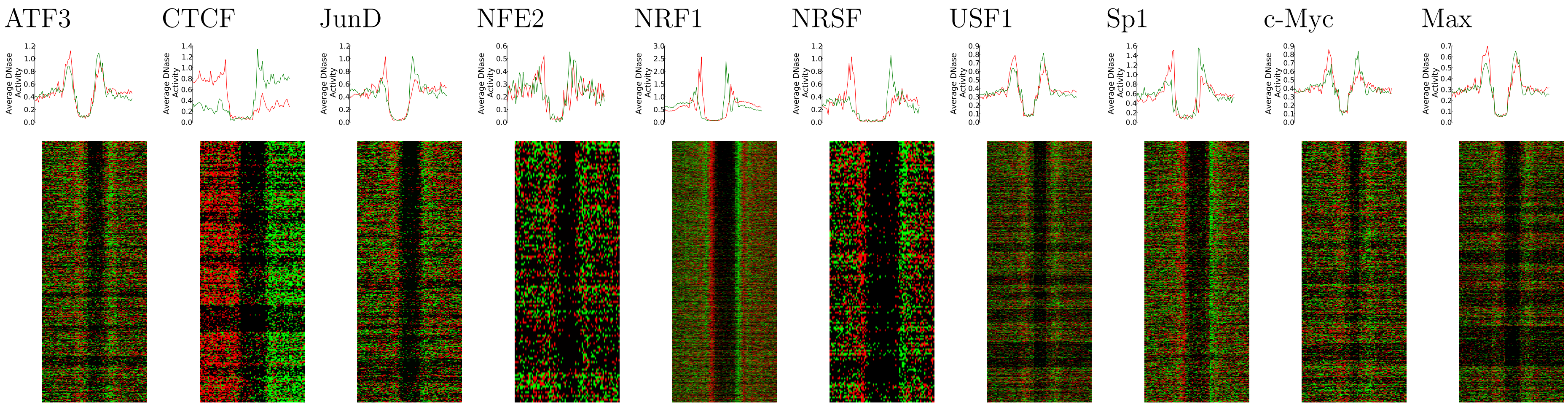

Figure S5: DNase-seq cleavage imbalance is less pronounced in DNase-seq data generated using the original single-hit DNase-seq library preparation protocol. Upper panels: DNase-seq cleavage patterns surrounding ChIP-seq verified binding sites in K562 cells with a Footprint Occupancy Score (FOS) of <0.95 illustrate the abundance of sequencing fragments aligning to the positive reference strand (red), and to the negative reference strand (green). Some transcription factors (CTCF, NRF1, NRSF) demonstrate cleavage imbalance patterns consistent with the double-hit protocol (Figure S4), whereas others (ATF3, JunD, NFE2) exhibit diminished strand imbalance. Lower panels: Heat map depicting cutting frequencies around the binding site. Binding sites are sorted from top to bottom in order of decreasing FOS.

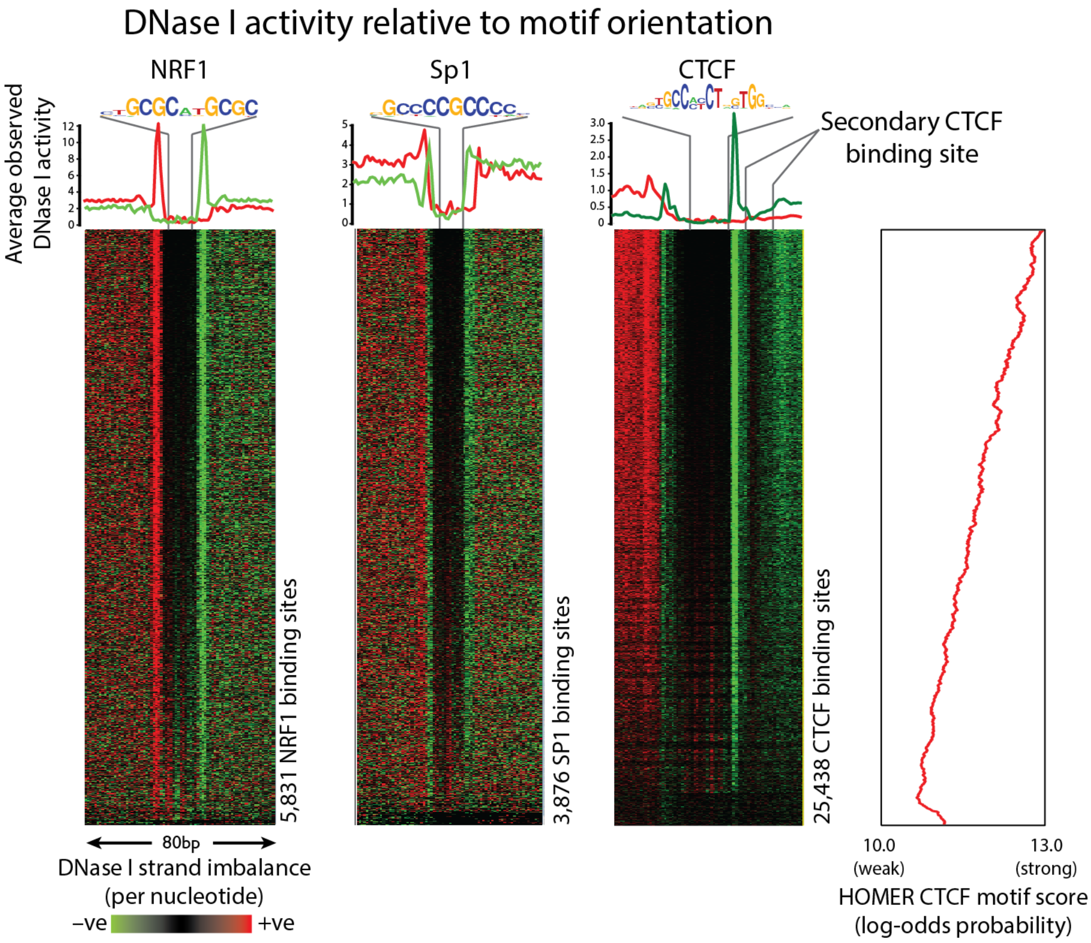

Figure S6: Heat maps show transcription factor specific DNase-seq cleavage patterns surrounding verified NRF1, Sp1, and CTCF binding sites. Here the data are oriented relative to motif strand which is indicated in the upper panels. Red indicates an excess of positive strand (with respect to motif strand) cuts over negative strand (with respect to motif strand) cuts per nucleotide position, and green indicates an excess of negative strand (with respect to motif strand) cuts. Binding sites are sorted from top to bottom in order of decreasing Footprint Occupancy Score [5]. Note that alignment of the CTCF motifs in the same orientation reveals an additional region corresponding to a secondary motif for CTCF binding (resembling CTGCAG; [11, 8]) that is also protected. For CTCF, the HOMER motif scores are presented as a moving average on the right, highlighting the fact that decreasing footprint scores equate with decreasing motif scores. Additional sub-patterns of DNase I cleavage can also be seen within motifs with lower occupancy scores, perhaps reflecting inefficient binding of some specific individual DNA-binding domains when bound to sub-optimal motifs.

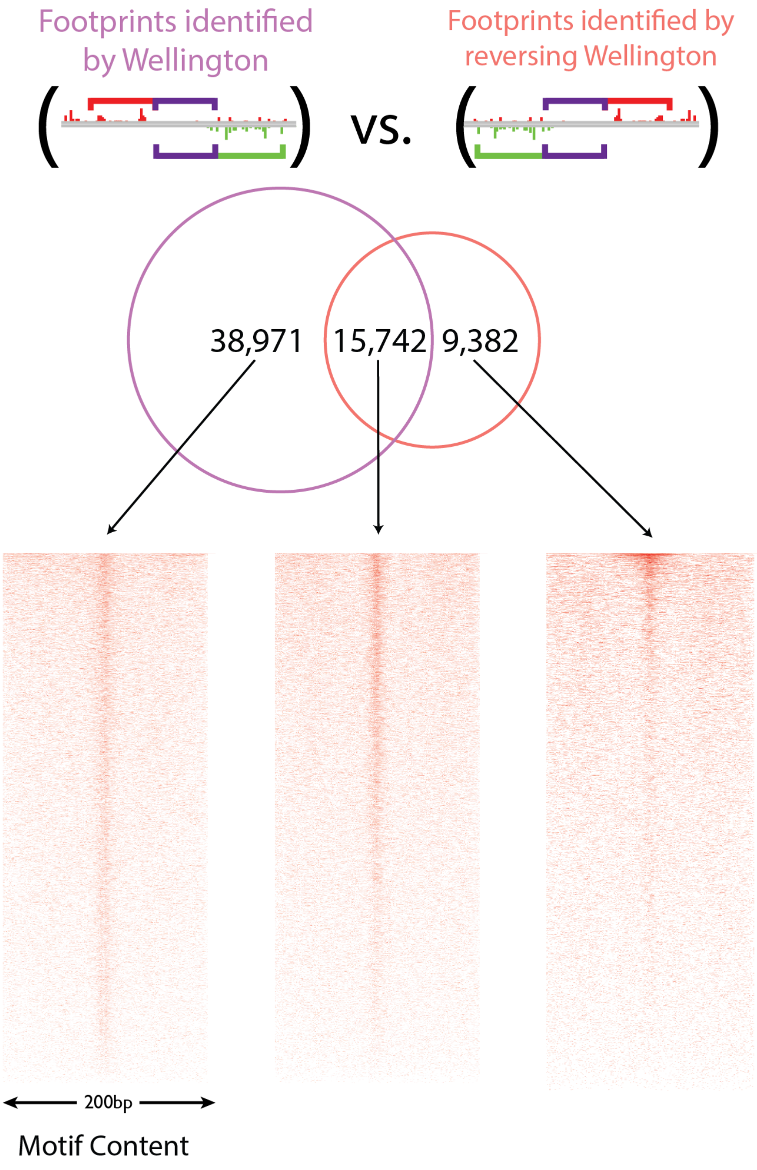

Figure S7: Motif content and motif location of Wellington and reverse Wellington footprints. Heatmaps of motif locations surrounding footprints identified by Wellington and reverse Wellington demonstratesthe depletion of motifs at the centre of reverse Wellington footprints.

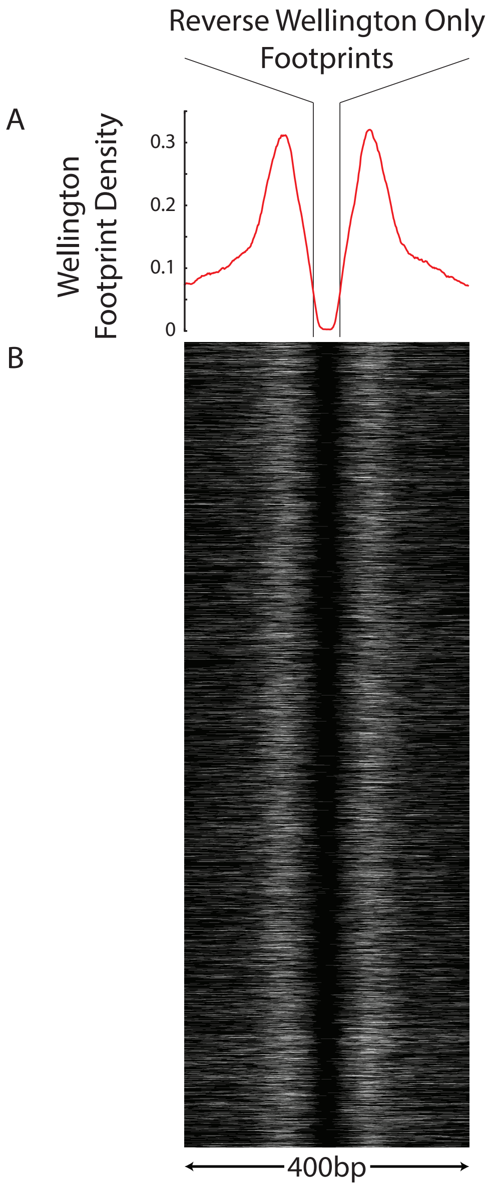

Figure S8: The majority of the 9,382 false positive footprints identified only by Reverse Wellington are located adjacent to or inbetween footprints identified by Wellington. (A) The distribution of Wellington footprints surrounding the 9,382 Reverse Wellington footprints, shown as the percentage of nucleotides at this position surrounding a Reverse Wellington Footprint which are found in a Wellington Footprint. (B) Heat map of footprints identified by Wellington centred on those only identified by Reverse wellington.

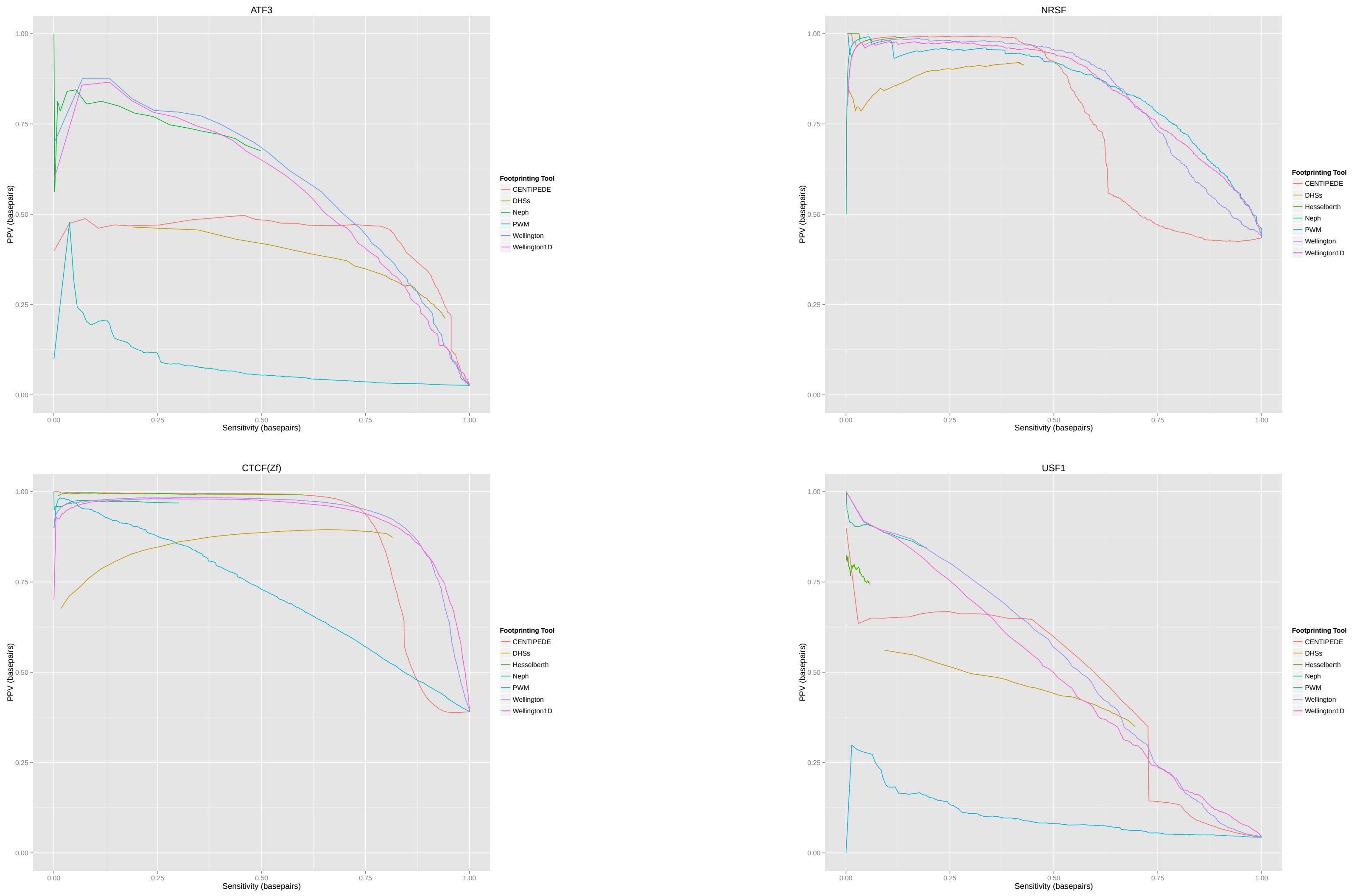

ATF3
NRSF
CTCF(Zf)
USF1
PPV (basepairs)
Sensitivity (basepairs)
0.00
0.25
0.50
0.75
1.00
Footprinting Tool
CENTIPEDE
DHSs
Hesselberth
Neph
PWM
Wellington
Wellington1D

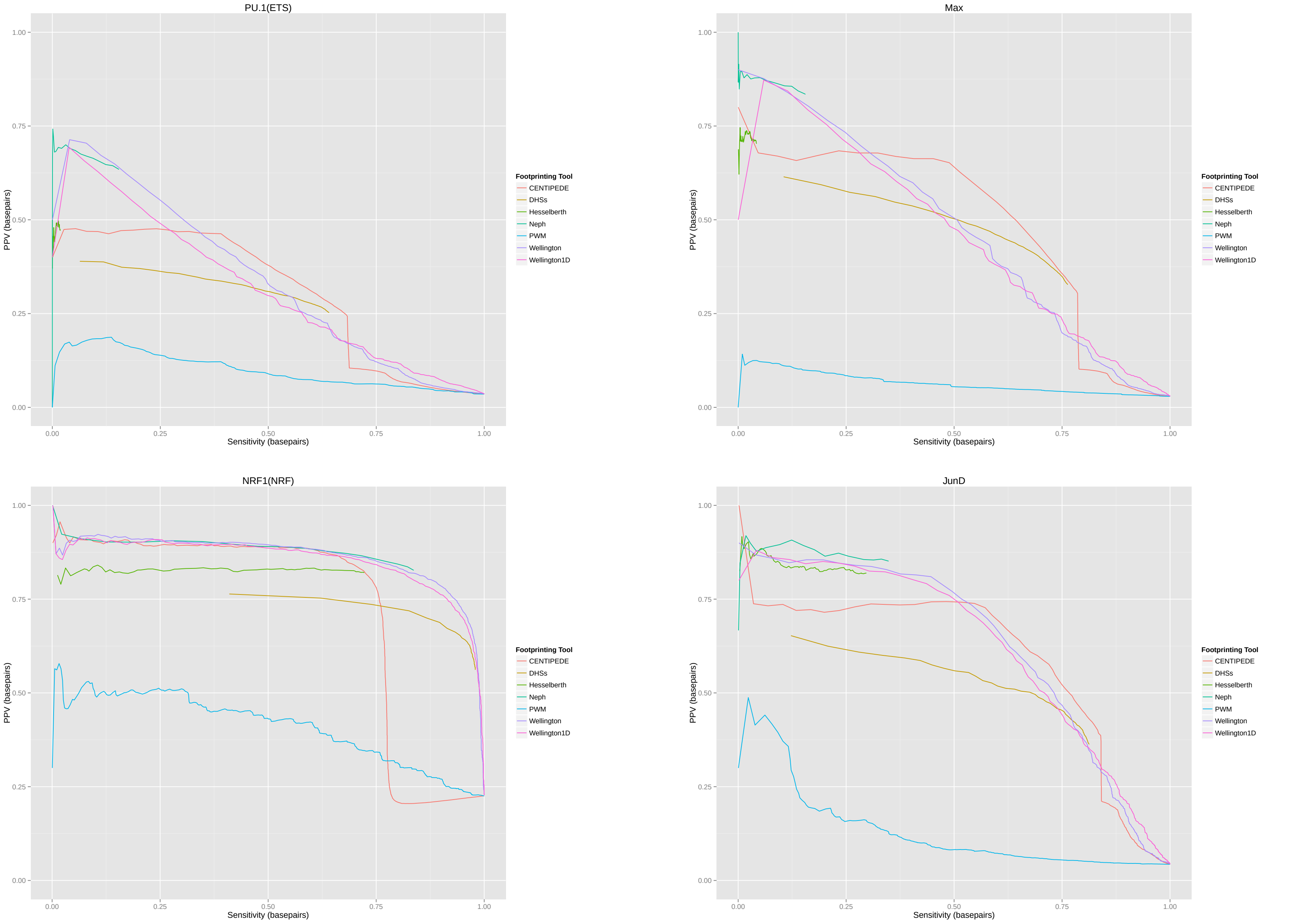
PU.1(ETS)
Max
NRF1(NRF)
JunD
PPV (basepairs)
Sensitivity (basepairs)
0.00
0.25
0.50
0.75
1.00
Footprinting Tool
CENTIPEDE
DHSs
Hesselberth
Neph
PWM
Wellington
Wellington1D

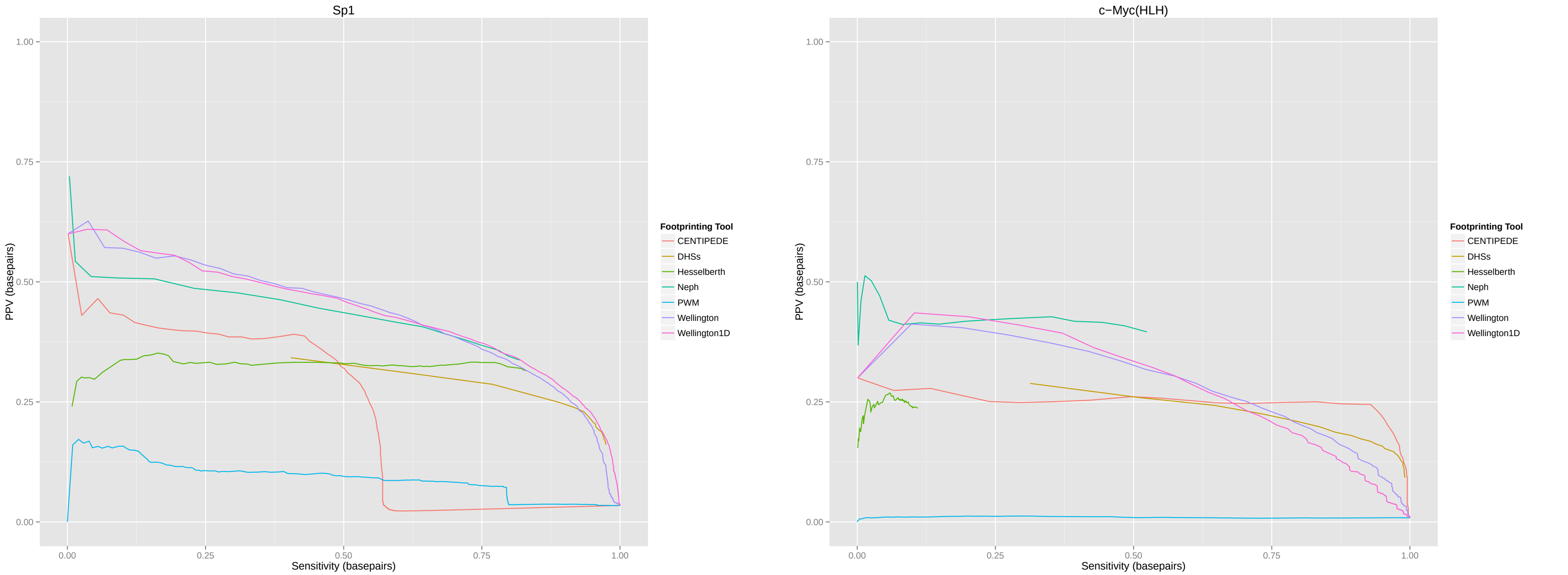

Figure S9: Positive Predictive Value of footprint predictions on ENCODE double-hit K562 DNase-seq data as a function of ChIP-seq sensitivity for 10 genomic transcription factor binding sites for Wellington, Wellington 1D, Neph et al., Hesselberth et al., Position Weight Matrices, DNase Hypersensitive Sites, and CENTIPEDE.

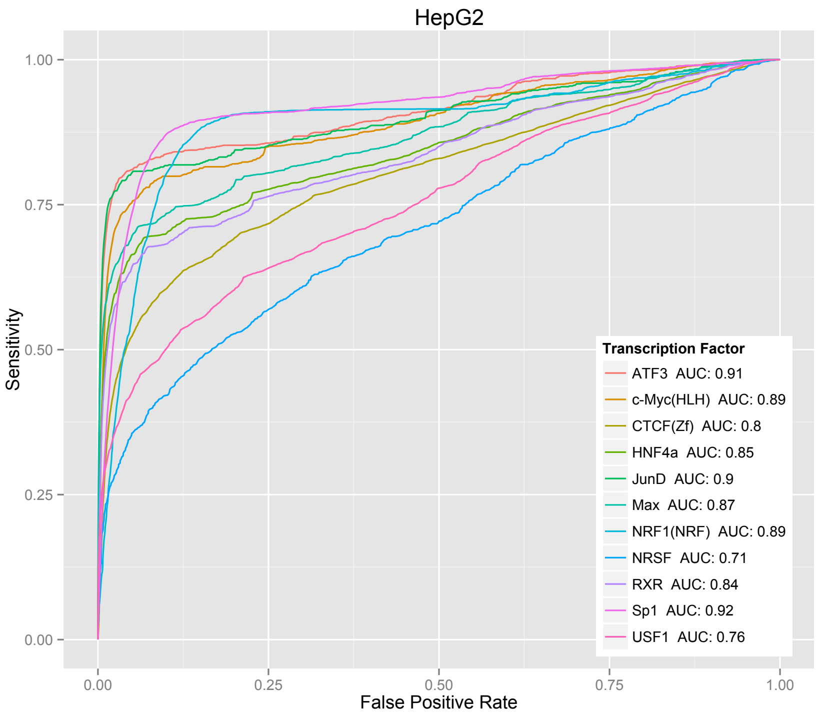

Figure S10: ROC analysis for 11 genomic transcription factor binding site predictions by Wellington using data from HepG2 cells

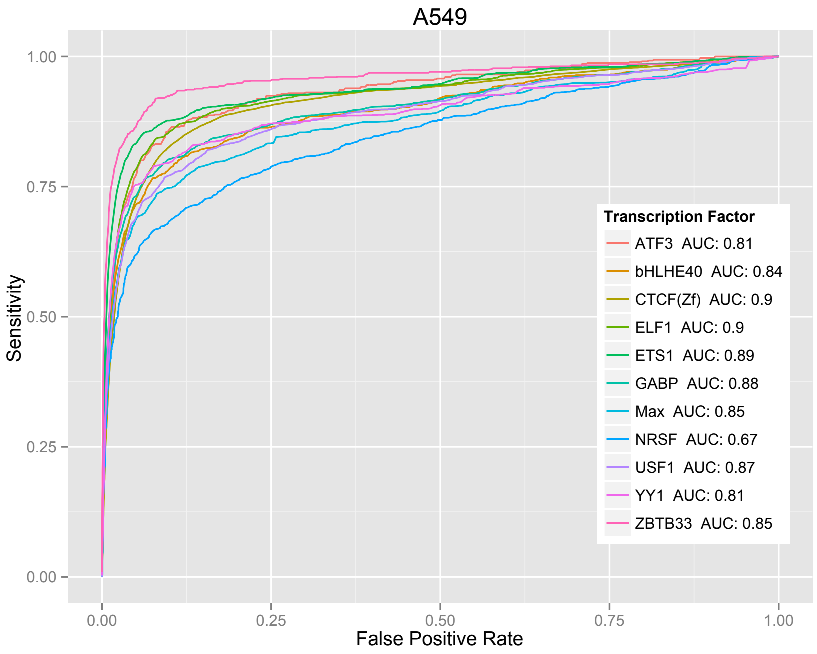

Figure S11: ROC analysis for 11 genomic transcription factor binding site predictions by Wellington using data from A549 cells

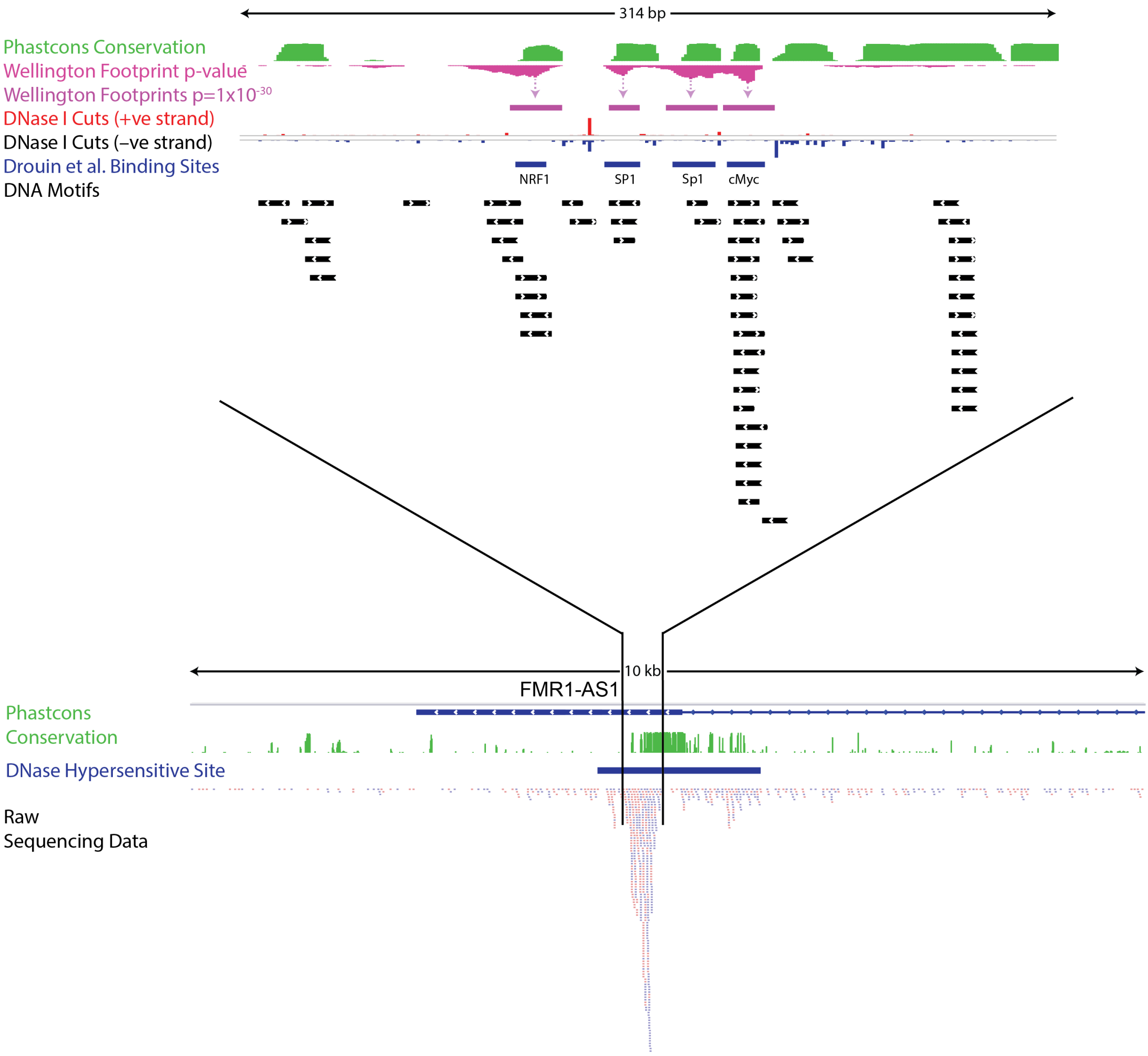

Figure S12: Footprints at the FMR1 promoter overlap with regions of high sequence conservation and improve over basic DNase-seq peak calling. Whilst phastcons conservation and DNA motifs overlap with known binding sites in this region, without footprinting, motif content alone is unable to predict bound locations. After applying the Wellington algorithm to the 1kb DNase hypersensitive site covering the FMR1 promoter, we produce footprints that align with the known protein-DNA interactions in this region without any off-target hits.

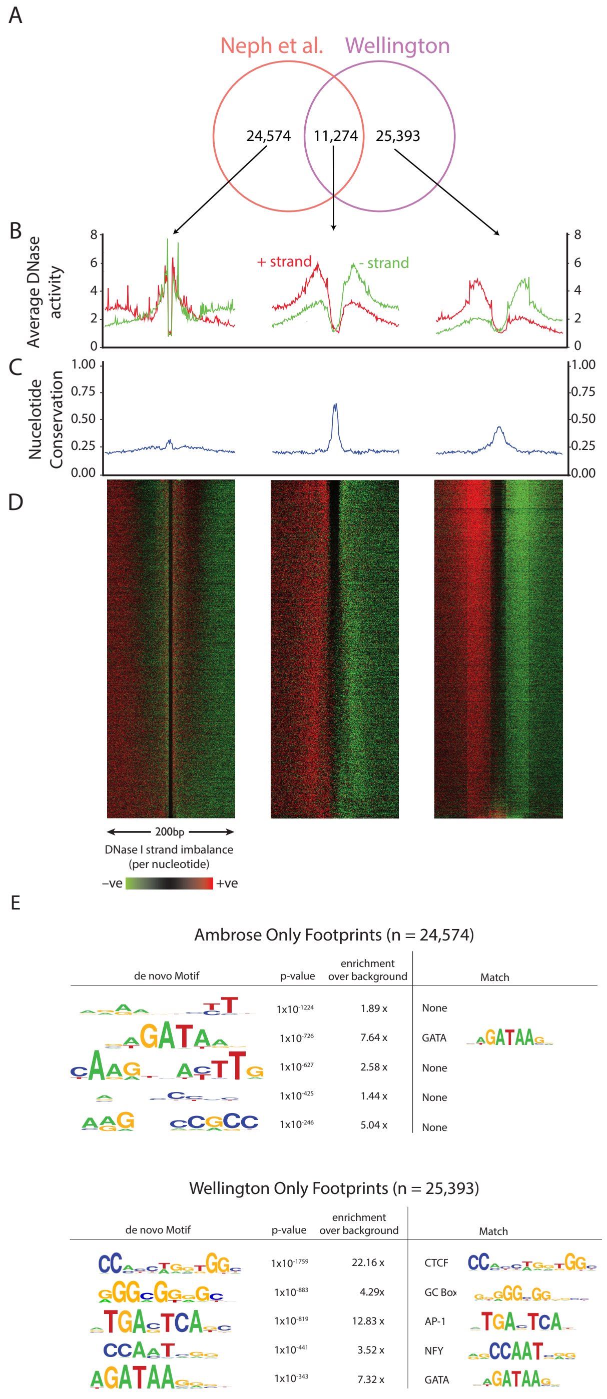

Figure S13: Footprints only identified by ENCODE [5] do not exhibit typical asymmetry. By comparing the 40,000 top scoring footprints for K562 cells from the ENCODE and the Wellington set, we observe that ENCODE exclusive footprints do not exhibit typical strand asymmetry identified in Figure 2 and have low average PhyloP conservation scores. *De novo* motif finding results show that Wellington footprints show more specific sequence logos, more enrichment over background, and more matches to known matrices.

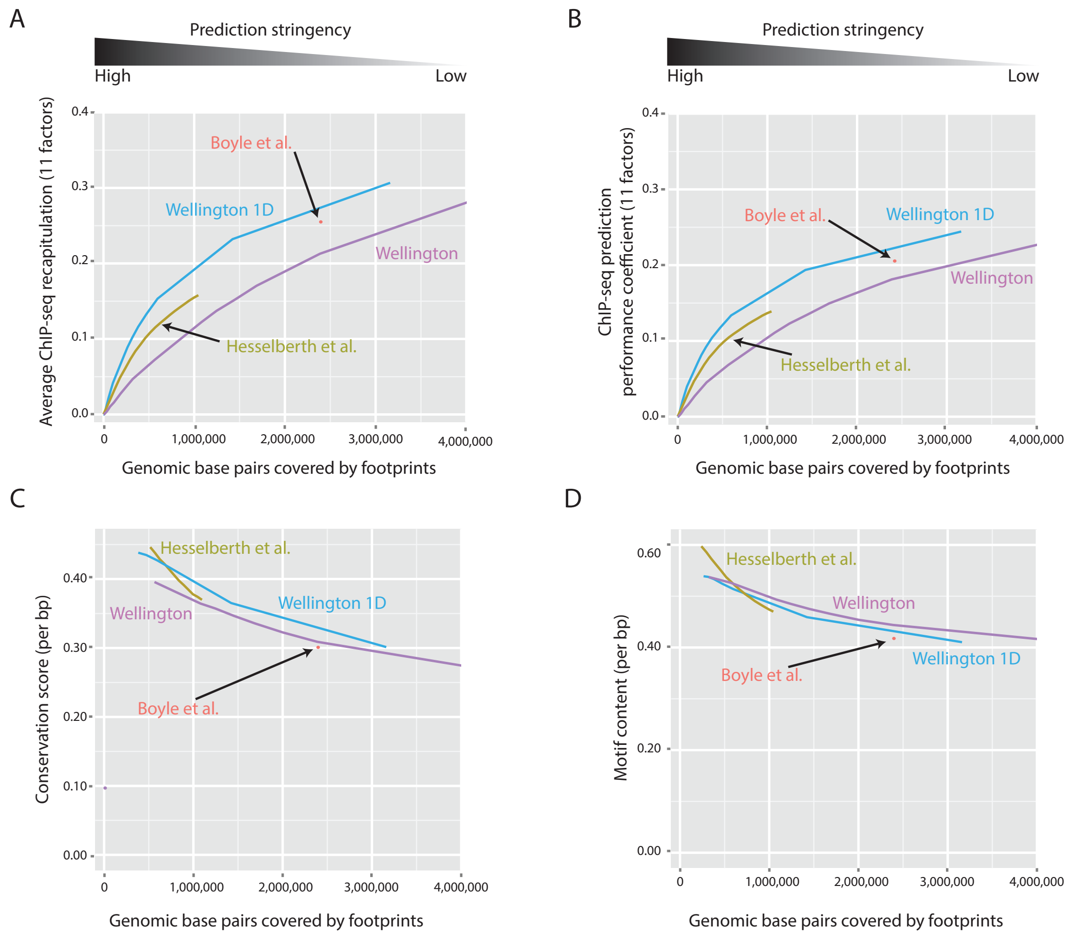

Figure S14: Wellington and Wellington 1D can also be used on DNase-seq data generated using the single-hit protocol. (A) Wellington is able to recapitulate a larger amount of ChIP-seq data than the predictions by Boyle et al. and Hesselberth et al. The horizontal axis shows the total number of base pairs in the genome that are covered by footprints at a given footprinting stringency, the vertical axis shows the average performance of these footprints in recapitulating binding sites found from ChIP-seq data for 11 transcription factors in K562 cells. (B) The nucleotide performance coefficients for these predictions Tompa:2005gx take numbers of false positives and false negatives into account and show a consistent finding compared to (A). (C, D) Wellington and Wellington 1D footprints have comparable conservation scores and motif content over a range of sensitivities, and with the available implementation, are able to detect more footprints than Hesselberth et al.

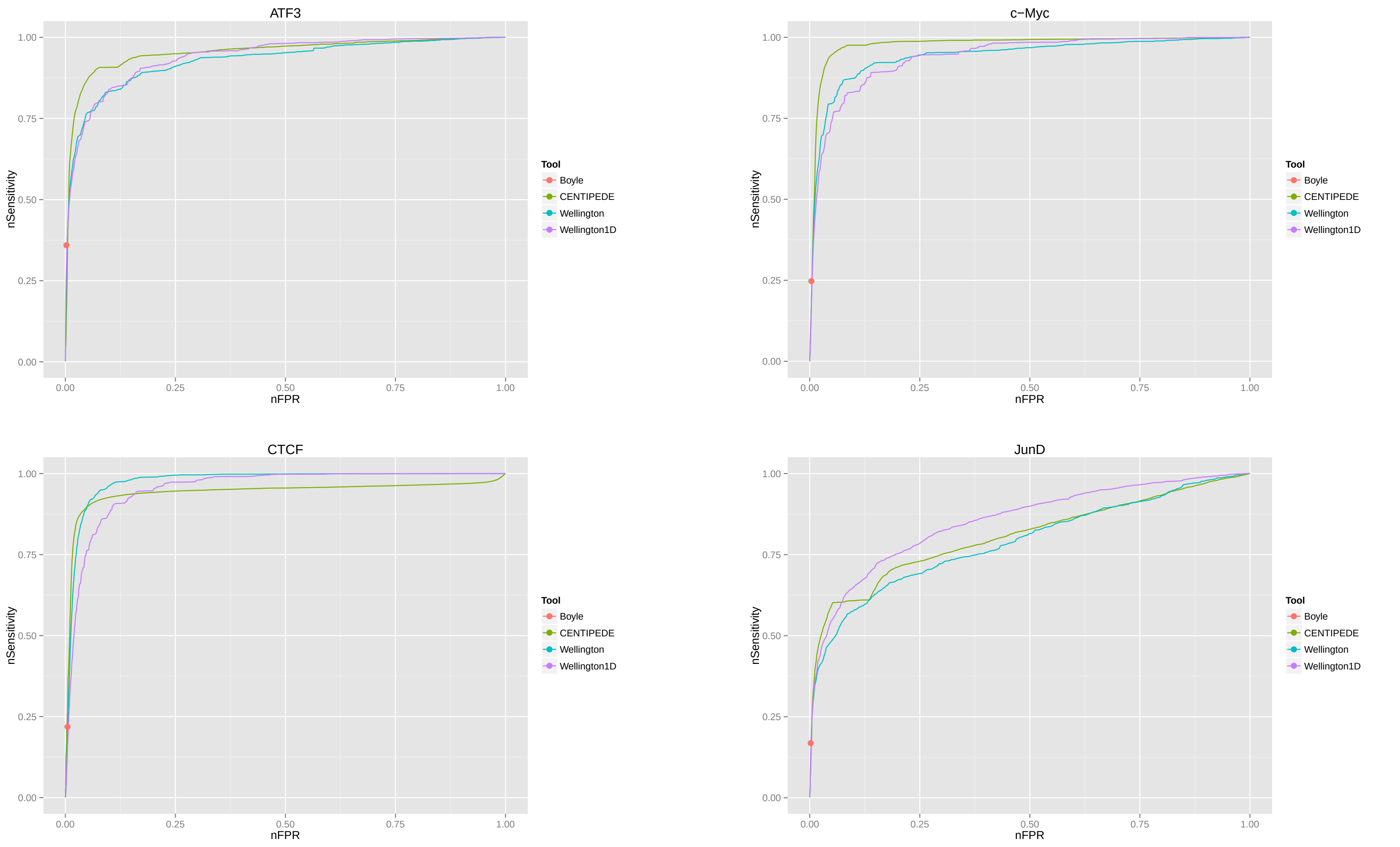
ATF3
c−Myc
CTCF
JunD
nSensitivity
nFPR
0.00
0.25
0.50
0.75
1.00
Tool
Boyle
CENTIPEDE
Wellington
Wellington1D

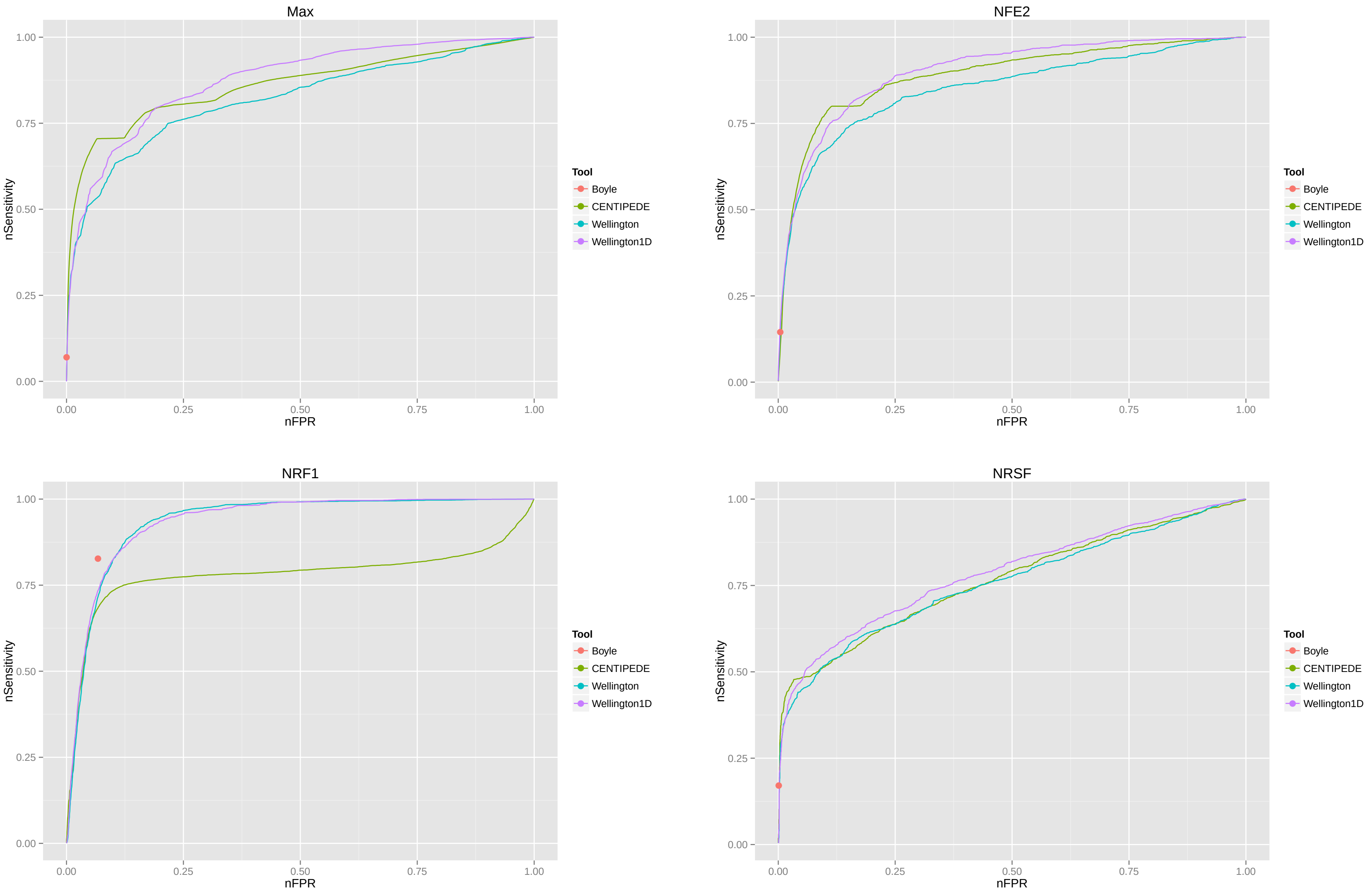
Max
NFE2
NRF1
NRSF
nSensitivity
nFPR
0.00
0.25
0.50
0.75
1.00
Tool
Boyle
CENTIPEDE
Wellington
Wellington1D

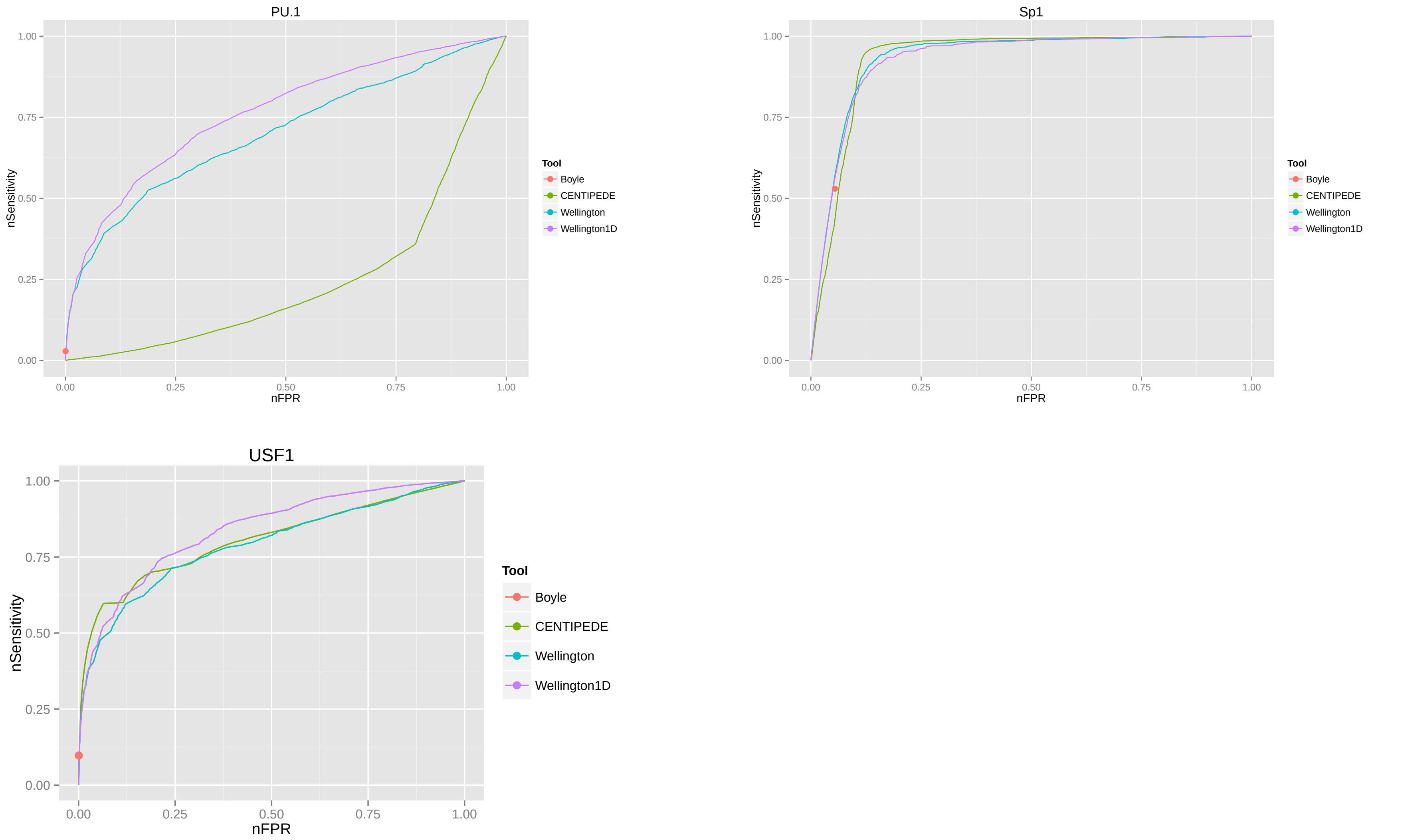

Figure S15: ROC analysis for Wellington, Wellington 1D, Boyle et al., and CENTIPEDE for transcription factor binding site predictions using K562 DNase-seq data data generated by the original single-hit library preparation.

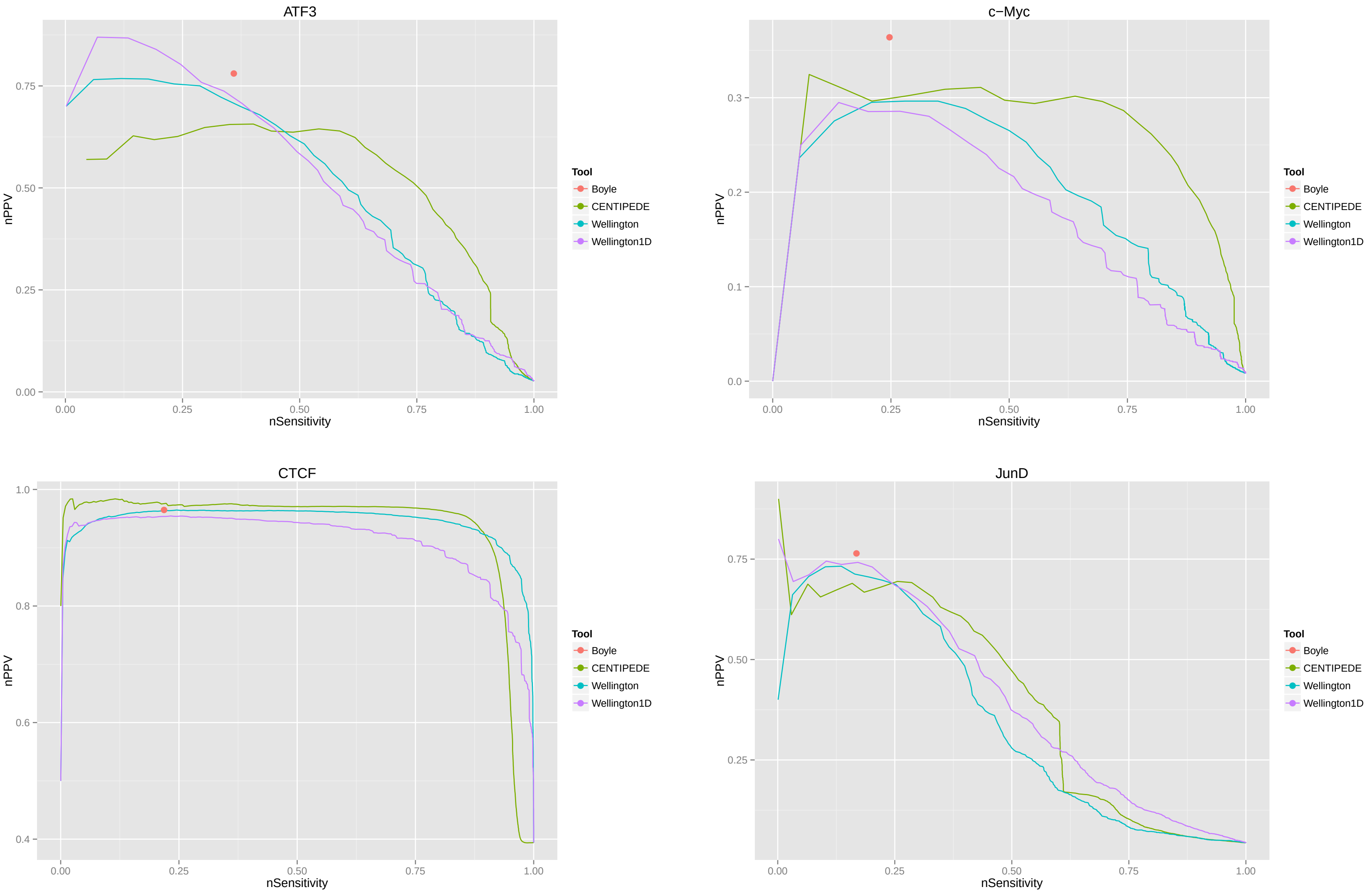

ATF3
c−Myc
CTCF
JunD
nPPV
nSensitivity
0.00
0.25
0.50
0.75
1.00
0.0
0.1
0.2
0.3
0.4
0.6
0.8
1.0
Tool
Boyle
CENTIPEDE
Wellington
Wellington1D

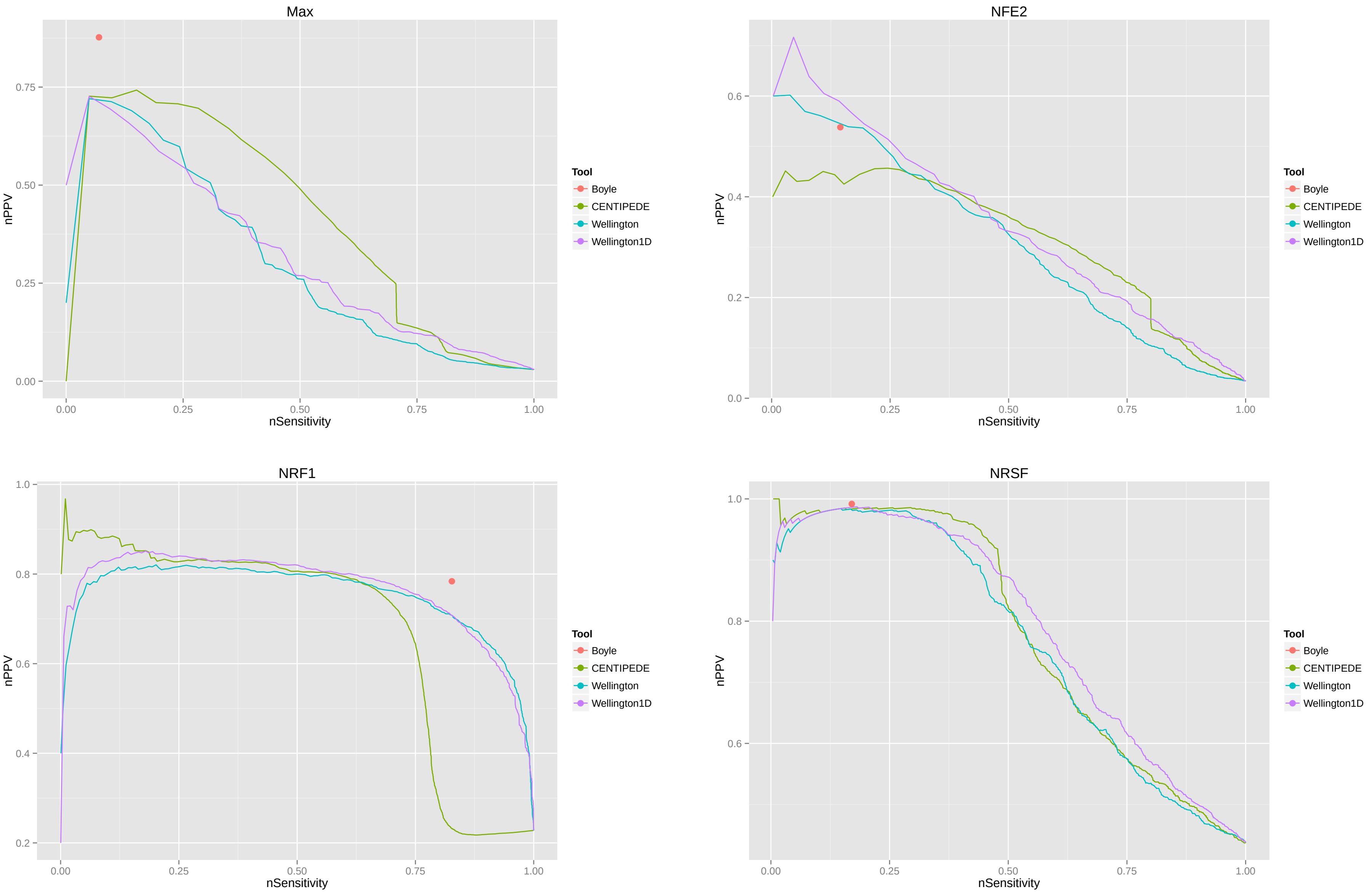
Max
NFE2
NRF1
NRSF
nPPV
nSensitivity
0.00
0.25
0.50
0.75
1.00
0.0
0.2
0.4
0.6
0.8
1.0
Tool
Boyle
CENTIPEDE
Wellington
Wellington1D

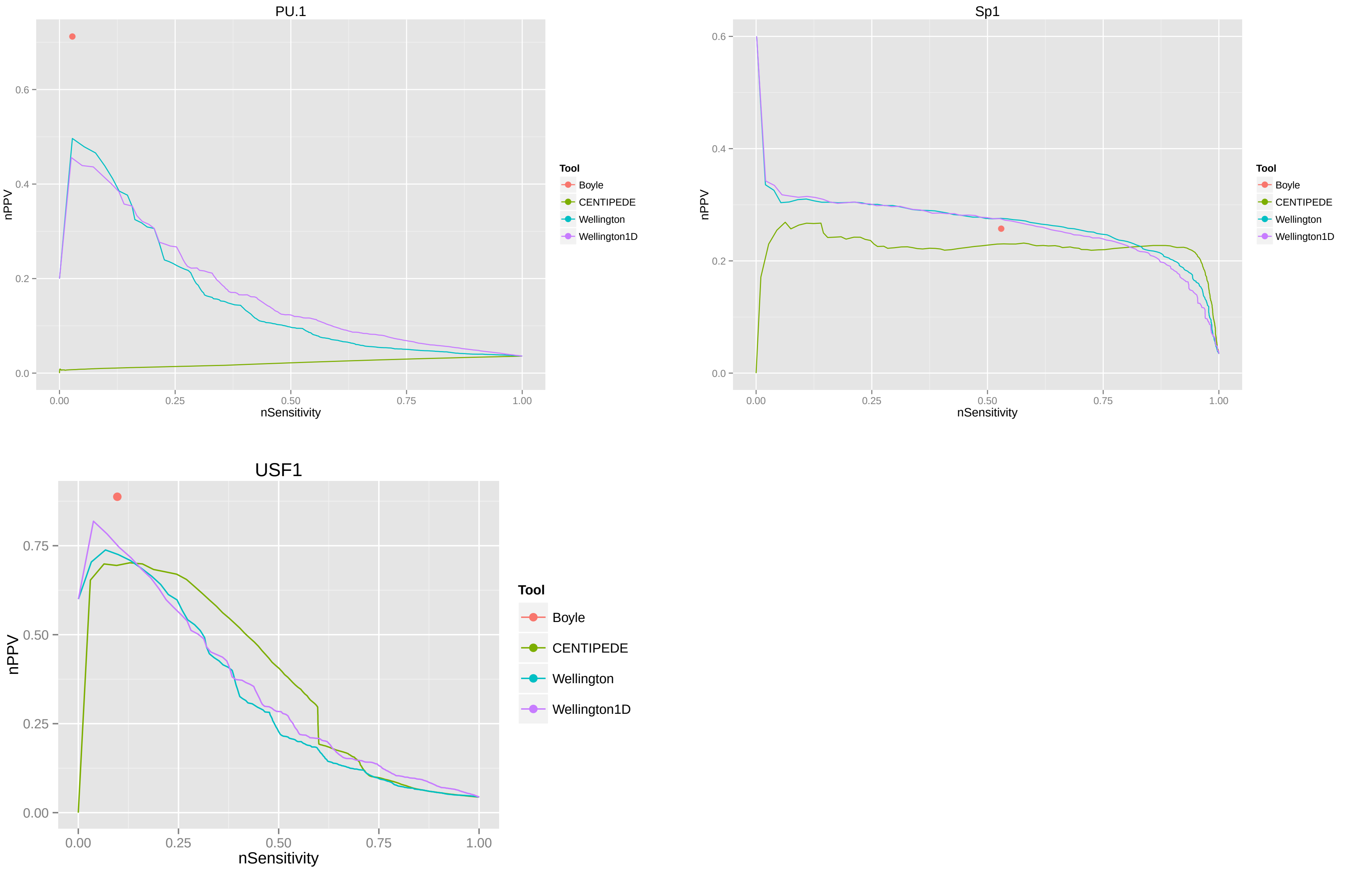

Figure S16: Positive Predictive Value of footprint predictions on K562 DNase-seq data data generated by the original single-hit library preparation as a function of ChIP-seq sensitivity for 11 genomic transcription factor binding for Wellington, Wellington 1D, Boyle et al. and CENTIPEDE.

References

[1] Peter J Sabo, Michael S Kuehn, Robert Thurman, Brett E Johnson, Ericka M Johnson, Hua Cao, Man Yu, Elizabeth Rosenzweig, Jeff Goldy, Andrew Haydock, Molly Weaver, Anthony Shafer, Kristin Lee, Fidencio Neri, Richard Humbert, Michael A Singer, Todd A Richmond, Michael O Dorschner, Michael McArthur, Michael Hawrylycz, Roland D Green, Patrick A Navas, William S Noble, and John A Stamatoyannopoulos. Genome-scale mapping of DNase I sensitivity in vivo using tiling DNA microarrays. *Nature Methods*, 3(7):511–518, July 2006.

[2] Alan P Boyle, Sean Davis, Hennady P Shulha, Paul Meltzer, Elliott H Margulies, Zhiping Weng, Terrence S Furey, and Gregory E Crawford. High-resolution mapping and characterization of open chromatin across the genome. *Cell*, 132(2):311–322, January 2008.

[3] Jay R Hesselberth, Xiaoyu Chen, Zhihong Zhang, Peter J Sabo, Richard Sandstrom, Alex P Reynolds, Robert E Thurman, Shane Neph, Michael S Kuehn, William S Noble, Stanley Fields, and John A Stamatoyannopoulos. Global mapping of protein-DNA interactions in vivo by digital genomic footprinting. *Nature Methods*, 6(4):283–289, April 2009.

[4] Peter N Cockerill. Structure and function of active chromatin and DNase I hypersensitive sites. *The FEBS journal*, 278(13):2182–2210, July 2011.

[5] Shane Neph, Jeff Vierstra, Andrew B Stergachis, Alex P Reynolds, Eric Haugen, Benjamin Vernot, Robert E Thurman, Sam John, Richard Sandstrom, Audra K Johnson, Matthew T Maurano, Richard Humbert, Eric Rynes, Hao Wang, Shinny Vong, Kristen Lee, Daniel Bates, Morgan Diegel, Vaughn Roach, Douglas Dunn, Jun Neri, Anthony Schafer, R Scott Hansen, Tanya Kutyavin, Erika Giste, Molly Weaver, Theresa Canfield, Peter Sabo, Miaohua Zhang, Gayathri Balasundaram, Rachel Byron, Michael J MacCoss, Joshua M Akey, M A Bender, Mark Groudine, Rajinder Kaul, and John A Stamatoyannopoulos. An expansive human regulatory lexicon encoded in transcription factor footprints. *Nature*, 489(7414):83–90, September 2012.

[6] Hashem Koohy, Thomas A Down, and Tim J Hubbard. Chromatin Accessibility

Data Sets Show Bias Due to Sequence Specificity of the DNase I Enzyme. *PloS one*, 8(7):e69853, 2013.

[7] Roger Pique-Regi, Jacob F Degner, Athma A Pai, Daniel J Gaffney, Yoav Gilad, and Jonathan K Pritchard. Accurate inference of transcription factor binding from DNA sequence and chromatin accessibility data. *Genome Research*, 21(3):447–455, March 2011.

[8] Alan P Boyle, Lingyun Song, Bum-Kyu Lee, Darin London, Damian Keefe, Ewan Birney, Vishwanath R Iyer, Gregory E Crawford, and Terrence S Furey. High-resolution genome-wide in vivo footprinting of diverse transcription factors in human cells. *Genome Research*, 21(3):456–464, March 2011.

[9] Sven Heinz, Christopher Benner, Nathanael Spann, Eric Bertolino, Yin C Lin, Peter Laslo, Jason X Cheng, Cornelis Murre, Harinder Singh, and Christopher K Glass. Simple combinations of lineage-determining transcription factors prime cis-regulatory elements required for macrophage and B cell identities. *Molecular cell*, 38(4):576–589, May 2010.

[10] Martin Tompa, Nan Li, Timothy L Bailey, George M Church, Bart De Moor, Eleazar Eskin, Alexander V Favorov, Martin C Frith, Yutao Fu, W James Kent, Vsevolod J Makeev, Andrei A Mironov, William Stafford Noble, Giulio Pavesi, Graziano Pesole, Mireille Régnier, Nicolas Simonis, Saurabh Sinha, Gert Thijs, Jacques van Helden, Mathias Vandenbogaert, Zhiping Weng, Christopher Workman, Chun Ye, and Zhou Zhu. Assessing computational tools for the discovery of transcription factor binding sites. *Nature biotechnology*, 23(1):137–144, January 2005.

[11] Sarion R Bowers, Fabio Mirabella, Fernando J Calero-Nieto, Stephanie Valeaux, Suzana Hadjur, Euan W Baxter, Matthias Merkenschlager, and Peter N Cockerill. A conserved insulator that recruits CTCF and cohesin exists between the closely related but divergently regulated interleukin-3 and granulocyte-macrophage colony-stimulating factor genes. *Molecular and cellular biology*, 29(7):1682–1693, April 2009.
